# The hepatic integrated stress response suppresses the somatotroph axis to control liver damage in nonalcoholic fatty liver disease

**DOI:** 10.1016/j.celrep.2022.111803

**Published:** 2022-12-13

**Authors:** Rika Ohkubo, Wei-Chieh Mu, Chih-Ling Wang, Zehan Song, Marine Barthez, Yifei Wang, Nathaniel Mitchener, Rasul Abdullayev, Yeong Rim Lee, Yuze Ma, Megan Curtin, Suraj Srinivasan, Xingjia Zhang, Fanghan Yang, Peter H. Sudmant, Angela Oliveira Pisco, Norma Neff, Cole M. Haynes, Danica Chen

**Affiliations:** 1 Metabolic Biology Graduate Program, University of California, Berkeley, Berkeley, CA 94720, USA; 2 Department of Nutritional Sciences and Toxicology, University of California, Berkeley, Berkeley, CA 94720, USA; 3 Endocrinology Graduate Program, University of California, Berkeley, Berkeley, CA 94720, USA; 4 Department of Integrative Biology, University of California, Berkeley, Berkeley, CA 94720, USA; 5 Center for Computational Biology, University of California, Berkeley, Berkeley, CA 94720, USA; 6 Chan Zuckerberg Biohub, San Francisco, CA, USA; 7 Department of Molecular, Cell and Cancer Biology, UMass-Chan Medical School, Worcester, MA 01605, USA; 8 Present address: MSD K.K., Kitanomaru Square, 1-13-12 Kudan-kita, Chiyoda-Ku, Tokyo 102-8667, Japan; 9 These authors contributed equally; 10 Lead contact

## Abstract

Nonalcoholic fatty liver disease (NAFLD) can be ameliorated by calorie restriction, which leads to the suppressed somatotroph axis. Paradoxically, the suppressed somatotroph axis is associated with patients with NAFLD and is correlated with the severity of fibrosis. How the somatotroph axis becomes dysregulated and whether the repressed somatotroph axis impacts liver damage during the progression of NAFLD are unclear. Here, we identify a regulatory branch of the hepatic integrated stress response (ISR), which represses the somatotroph axis in hepatocytes through ATF3, resulting in enhanced cell survival and reduced cell proliferation. In mouse models of NAFLD, the ISR represses the somatotroph axis, leading to reduced apoptosis and inflammation but decreased hepatocyte proliferation and exacerbated fibrosis in the liver. NAD^+^ repletion reduces the ISR, rescues the dysregulated somatotroph axis, and alleviates NAFLD. These results establish that the hepatic ISR suppresses the somatotroph axis to control cell fate decisions and liver damage in NAFLD.

## In brief

Ohkubo et al. show that hepatic ER stress results in the suppression of the somatotroph axis, which controls liver damage in nonalcoholic fatty liver disease by preventing hepatocyte death and proliferation, resulting in reduced inflammation but increased fibrosis. This study has important implications in treating this prevalent metabolic disease.

## Graphical Abstract

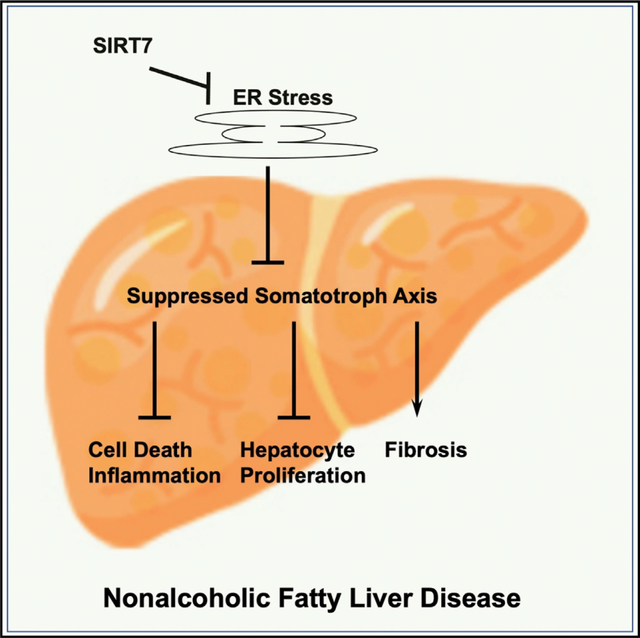

## INTRODUCTION

The current challenges in developing therapeutics against nonalcoholic fatty liver disease (NAFLD) reflect its complex nature, raising the question of whether the solution requires a combination of drugs. NAFLD can be ameliorated by calorie restriction, which leads to the suppressed growth hormone/insulin-like growth factor-1 (IGF-1) somatotroph axis, a conserved regulator of lifespan that triggers the activation of the cellular protective program and the re-allocation of resources from growth to somatic preservation.^1–9^ Paradoxically, suppression of the somatotroph axis is associated with patients with NAFLD and, in particular, is correlated with the severity of fibrosis.^10–19^ Whether the somatotroph axis controls liver damage during the progression of NAFLD is unknown.

NAFLD begins with hepatosteatosis and can progress to nonalcoholic steatohepatitis (NASH) in response to endoplasmic reticulum (ER) stress.^20–22^ The integrated stress response (ISR) is a critical regulator of protein homeostasis at the cellular and organismal level to control the pathogenesis of complex diseases.^23^ Little is known about the connectivity of the ISR to other intracellular signaling networks to determine cell fate decisions and physiological output. The growth hormone/IGF-1 somatotroph axis includes the secretion of growth hormone from the somatotropes of the pituitary gland into the circulation and the subsequent stimulation of IGF-1 production, which is synthesized and secreted by the liver.^24^ While evidence is emerging that systemic ER stress induction leads to the suppressed somatotroph axis,^25^ whether hepatic ER stress regulates the somatotroph axis autonomously and the molecular mechanism underlying such regulation remain unexplored.

In this study, we show that hepatic ER stress suppresses the somatotroph axis autonomously through the transcription factor ATF3. We provide evidence that suppression of the somatotroph axis results in reduced apoptosis and inflammation but decreased hepatocyte proliferation and exacerbated fibrosis in the livers, offering explanations for the paradoxical observations that the suppressed somatotroph axis is associated with patients with NAFLD while calorie restriction suppresses the somatotroph axis and prevents the development of NAFLD at the early stage. Finally, we demonstrate the therapeutic implication of this regulatory pathway for NAFLD.

## RESULTS

### A mouse model of NAFLD with the suppressed somatotroph axis

**Figure 1.**
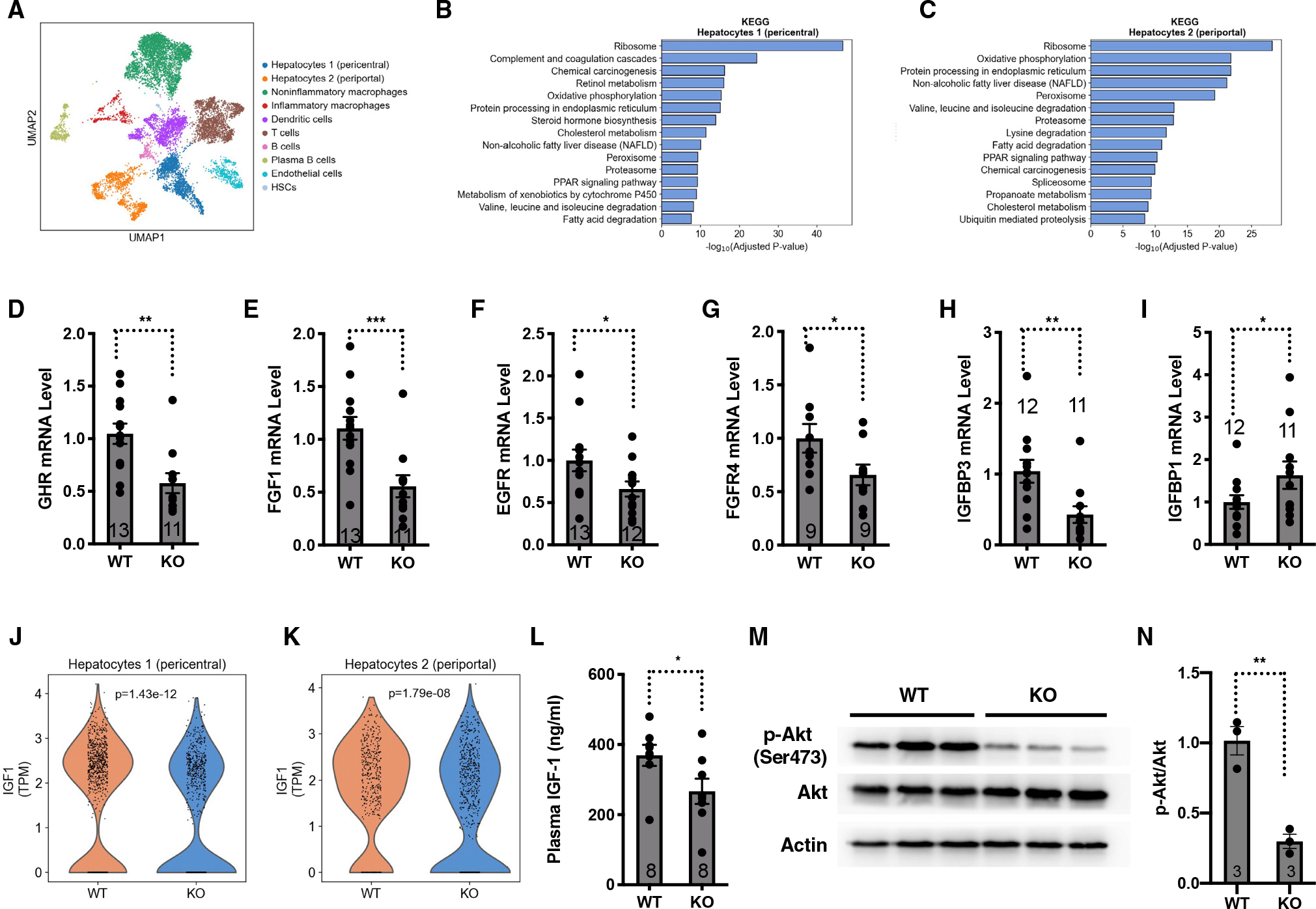
A mouse model of NAFLD with the suppressed somatotroph axis (A) Single-cell RNA sequencing of the livers of WT and *SIRT7*^−/−^ mice using the 10x Genomics Chromium platform. Uniform manifold approximation and projection (UMAP) clustering of single-cell transcriptomes (3,270 cells from WT and 8,340 cells from *SIRT7*^−/−^ mice) colored by cell type. n = 3 mice. (B and C) Pathway analysis for the biological function of differentially expressed genes in hepatocyte 1 (pericentral) and hepatocyte 2 (periportal) of the livers of WT and *SIRT7*^−/−^ mice. n = 3 mice. (D–I) Quantitative real-time PCR analyses for the mRNA levels of the indicated genes in the livers of *SIRT7*^−/−^ mice and wild-type controls. GAPDH was used as an internal control. n = 9–13 mice. (J and K) Violin plots comparing log-normalized expression values of IGF-1 in hepatocyte 1 (pericentral) and hepatocyte 2 (periportal) in the livers of WT and *SIRT7*^−/−^ mice. Each dot represents the gene expression levels in one cell. Wilcoxon rank-sum test. n = 3 mice. (L) ELISA quantification of plasma levels of IGF-1 in *SIRT7*^−/−^ mice and wild-type controls. n = 8 mice. (M and N) Western analyses (M) and quantification (N) of phosphorylated Akt in the livers of *SIRT7*^−/−^ mice and wild-type controls. n = 3 mice. Error bars represent standard errors. *p < 0.05; **p < 0.01; ***p < 0.001. See also Figure S1 and S2.

To investigate how ER stress and the ISR drive the progression of liver damage in NASH and avoid the confounding factors derived from the diets that are commonly used to induce NASH, we employed a mouse NASH model deficient in the histone deacetylase SIRT7 that develops spontaneous NASH resembling human fatty liver disease when fed a chow diet due to elevated ER stress.^26–28^ Single-cell RNA sequencing of the livers of wild-type and *SIRT7*^−/−^ mice using the 10x Genomics Chromium platform and the pathway analysis of differentially expressed genes showed that NAFLD genes were highly enriched in several cell populations (hepatocytes, macrophages, and plasma B cells) of *SIRT7*^−/−^ livers (Figures 1A–1C and S1A–S1E; Data S1; Table S1), validating the NAFLD mouse model.

Microarray analysis of the livers of wild-type and *SIRT7*^−/−^ mice showed that a number of genes in the somatotroph growth axis and other mitogenic signals were differentially expressed between these two genotypes. The expression of several pro-growth factors, such as growth hormone receptor (GHR), fibroblast growth factor 1 (FGF1), epidermal growth factor receptor (EGFR), FGF receptor 4 (FGFR4), was suppressed in the livers of *SIRT7*^−/−^ mice (Figure S2; Data S2). IGF-binding proteins that positively correlate with the level of IGF-1, such as IGF-binding protein 3 (IGFBP3) and IGF-binding protein acid labile (IGFALS), were also suppressed in the livers of *SIRT7*^−/−^ mice, while IGF-binding proteins that generally inhibit the activity of IGF-1, such as IGFBP1, were upregulated. This pattern of gene expression changes in the livers of *SIRT7*^−/−^ mice and wild-type littermates was confirmed by quantitative real-time PCR (Figures 1D–1I). The analysis of the single-cell RNA sequencing data for the livers of wild-type and *SIRT7*^−/−^ mice revealed that the expression of the somatotroph gene IGF-1 was reduced in the hepatocytes of *SIRT7*^−/−^ liver (Figures 1J and 1K).

Circulating IGF-1 levels in *SIRT7*^−/−^ mice were significantly lower than their wild-type counterparts (Figure 1L). Consistent with reduced levels of blood IGF-1, the IGF-1 signaling was decreased in the livers of *SIRT7*^−/−^ mice, as evidenced by reduced phosphorylation of Akt (Figure 1M and 1N). The downregulation of the growth hormone/IGF-1 somatotroph axis in the livers of *SIRT7*^−/−^ mice is consistent with their post-natal growth retardation.^27,28^ Together, these data indicate suppressed somatotroph axis in *SIRT7*^−/−^ mice. This mouse model was therefore used to investigate how the somatotroph axis becomes dysregulated in NAFLD and to dissect the role of the somatotroph axis in the progression of NASH.

### Hepatic ER stress suppresses the somatotroph axis autonomously

**Figure 2.**
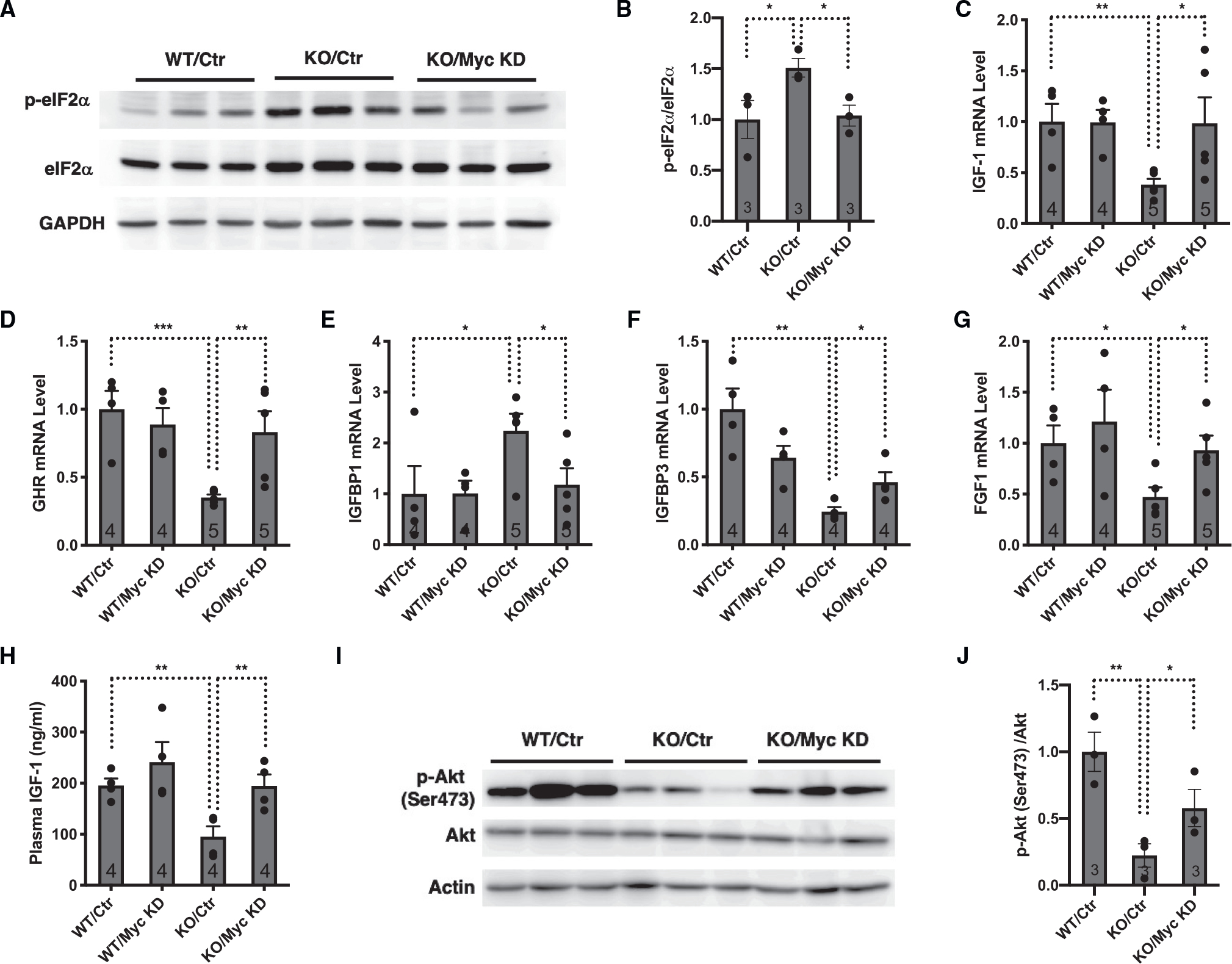
Hepatic ER stress suppresses the somatotroph axis autonomously Comparison of wild-type and *SIRT7*^−/−^ mice with or without Myc knockdown mediated by AAV8-mediated gene delivery. Mice were analyzed 4 weeks after viral infection. (A and B) Western analyses (A) and quantification (B) for phosphorylated eIF2α in the livers. n = 3 mice. (C–G) Quantitative real-time PCR analyses for the mRNA levels of the indicated genes in the livers. GAPDH was used as an internal control. n = 4–5 mice. (H) ELISA analyses of plasma levels of IGF-1. n = 4 mice. (I and J) Western analyses (I) and quantification (J) for phosphorylated Akt in the livers. n = 3 mice. Error bars represent standard errors. *p < 0.05; **p < 0.01; ***p < 0.001. See also Figure S3.

SIRT7 deficiency results in constitutive hepatic ER stress.^27^ We asked whether suppression of the somatotroph axis in *SIRT7*^−/−^ mice could result from hepatic ER stress and the induction of the ISR autonomously. SIRT7 suppresses ER stress by repressing the activity of the transcription factor Myc and reducing the expression of translation machinery.^27^ Consistently, the analysis of the single-cell RNA sequencing data for the livers of wild-type and *SIRT7*^−/−^ mice showed that ribosome genes were among the most significant changes in various cell types of the liver associated with SIRT7 expression (Figure 1B, 1C, S1D, and S1E). We knocked down the expression of Myc in the livers of *SIRT7*^−/−^ mice via adeno-associated virus 8 (AAV8)-mediated gene transfer. Myc inactivation repressed the ISR in the livers of *SIRT7*^−/−^ mice as evidenced by the levels of phosphorylation of eIF2α (Figures 2A and 2B). Myc inactivation also rescued the expression of genes in the somatotroph axis that were dysregulated in the livers of *SIRT7*^−/−^ mice (Figures 2C–2G), increased the plasma levels of IGF-1 (Figure 2H), and enhanced the hepatic IGF-1 signaling (Figures 2I and 2J), consistent with the suppression of the somatotroph axis by the hepatic ISR autonomously. Furthermore, treatment of hepatocytes with ER stress inducers thapsigargin or tunicamycin resulted in reduced expression of genes in the somatotroph axis (Figures S3A–S3E). Together, these data suggest that hepatic ER stress and the ISR induction are sufficient to trigger the response in the somatotroph axis autonomously.

### Hepatic ER stress and the ISR suppress the somatotroph axis by inducing ATF3

**Figure 3.**
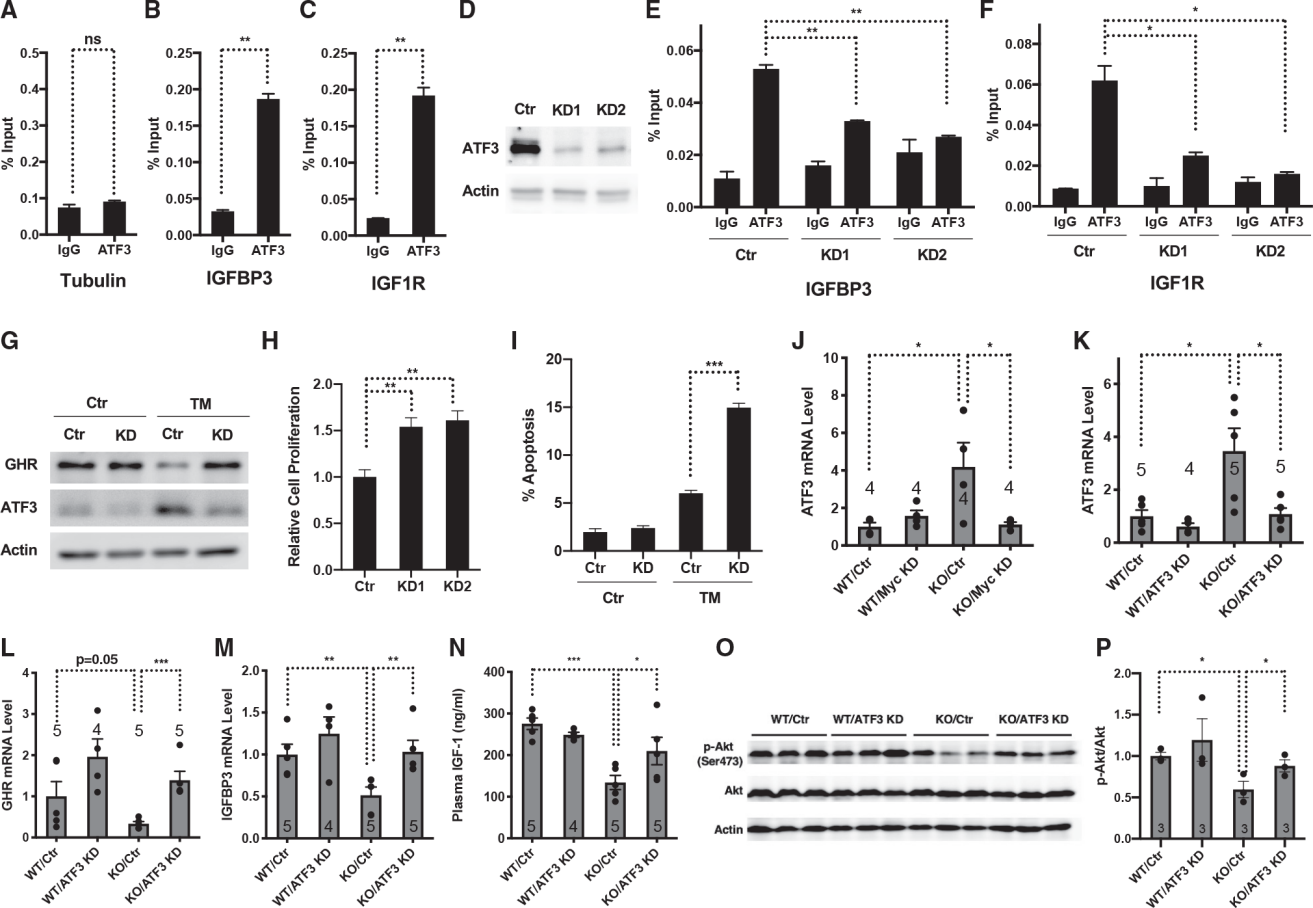
Hepatic ER stress and the ISR suppress the somatotroph axis by inducing ATF3 (A–C) ChIP with ATF3 antibody followed by quantitative real-time PCR showing ATF3 occupancy at the gene promoters of IGFBP3 and IGF1R in Hepa 1–6 cells. Tubulin was used as a negative control. n = 2. (D) Western blots showing ATF3 expression in stable ATF3 knockdown Hepa 1–6 cells using shRNA. (E and F) ChIP with ATF3 antibody followed by quantitative real-time PCR showing reduced ATF3 occupancy at the gene promoters of IGFBP3 and IGF1R in ATF3 knockdown Hepa 1–6 cells. n = 2. (G) Western analyses of GHR and ATF3 in control and ATF3 knockdown Hepa 1–6 cells with or without tunicamycin induction. (H) Proliferation of stable ATF3 knockdown Hepa 1–6 cells and control cells. n = 3. (I) Annexin V staining of ATF3 knockdown and control Hepa 1–6 cells with or without tunicamycin induction was analyzed with flow cytometry. n = 3. (J) Quantitative real-time PCR analyses of mRNA levels of ATF3 in the livers of *SIRT7*^−/−^ mice and wild-type mice with or without Myc knockdown mediated by AAV8-mediated gene delivery. Mice were analyzed 4 weeks after viral infection. n = 4 mice. (K–P) Comparison of *SIRT7*^−/−^ mice and wild-type mice with or without ATF3 knockdown mediated by AAV8-mediated gene delivery. Mice were analyzed 4 weeks after viral infection. (K–M) Quantitative real-time PCR analyses of mRNA levels of indicated genes in the livers. GAPDH was used as an internal control. n = 4–5 mice. (N) Elisa analyses of plasma levels of IGF-1. n=4–5 mice. (O and P) Western analyses (O) and quantification (P) for phosphorylated Akt in the livers. n = 3 mice. Error bars represent standard errors. *p < 0.05; **p < 0.01; ***p < 0.001; ns p > 0.05. See also Figure S4.

We next investigated how the hepatic ISR leads to the suppression of the somatotroph axis. ER stress elicits signaling transduction and stress response that allow the cells to restore protein homeostasis.^29^ Central to the ISR is the actions of the transcription factors ATF4 and ATF6. ATF3 is also induced by ER stress by a mechanism requiring eIF2 kinases and ATF4, although its role in stress response is obscure (Figures S4A and S4B and Jiang et al.^30^). We used the Harmonizome web portal, which is a collection of processed datasets to mine information related to genes and proteins,^31^ to determine whether the ER-stress-related transcription factors could regulate genes in the somatotroph axis. Chromatin immunoprecipitation (ChIP) sequencing data analyses revealed that ATF3 bound to the promotors or enhancers of a number of IGF-related genes (Figure S4C) and that ATF4 or ATF6 did not. The binding of ATF3 to the promoters of IGF-related genes was further confirmed by ChIP with an ATF3 antibody, followed by quantitative real-time PCR in parental hepatocytes (Figures 3A–3C) and mouse livers (Figures S4D–S4F), and was abrogated in ATF3 knockdown (KD) cells generated using two independent short hairpin RNAs (Figures 3D–3F). While treatment with the ER-stress-inducer tunicamycin reduced the expression of genes in the somatotroph axis, ATF3 inactivation blunted the effect (Figure 3G), suggesting that ER stress and the ISR induction repress the somatotroph axis in hepatocytes by inducing ATF3.

Suppression of the somatotroph axis leads to metabolic changes that shift energy usage from growth and proliferation to cellular protection in order to enhance stress resistance, a phenomenon termed hormesis.^1,3–8^ ATF3-mediated suppression of the somatotroph axis in response to ER stress and the ISR induction suggests that this branch of the ISR might prevent cell growth and proliferation while activating cellular protective programs and preventing cell death. ATF3 KD hepatocytes proliferated faster than control cells (Figure 3H) and exhibited increased apoptosis upon treatment with tunicamycin compared with control cells (Figure 3I). Together, these data suggest that ER stress and the ISR induce ATF3 to repress the somatotroph axis, resulting in reduced proliferation and improved survival of hepatocytes.

ATF3 is a member of the CREB family of basic leucine zipper transcription factors and functions both as a transcriptional activator or repressor.^32^ ATF3 is induced in the livers of a rat model of severe steatosis and human patients with NAFLD, correlative with the ER stress status.^33^ ATF3 was also induced in the livers of *SIRT7*^−/−^ mice (Figure 3J; Data S2). Myc inactivation in the livers of *SIRT7*^−/−^ mice via AAV8-mediated gene transfer suppressed the ISR (Figures 2A and 2B) and rescued the increased ATF3 expression (Figure 3J), consistent with the induction of ATF3 expression upon the hepatic ISR. To determine whether hepatic ISR results in suppression of the somatotroph axis due to the induction of ATF3, we knocked down the expression of ATF3 in the livers of *SIRT7*^−/−^ mice via AAV8-mediated gene transfer (Figure 3K). ATF3 inactivation in the livers of *SIRT7*^−/−^ mice rescued the dysregulated gene expression of the somatotroph axis (Figures 3L and 3M), in keeping with the binding of ATF3 to the promoters of IGF-related genes (Figures 3A–3C and S4C–S4F). ATF3 inactivation in the livers of *SIRT7*^−/−^ mice also increased the plasma levels of IGF-1 (Figure 3N) and the IGF-1 signaling (Figures 3O and 3P). Together, these data suggest that ATF3 mediates the hepatic ISR-induced repression of the somatotroph axis *in vivo*.

### Suppression of the somatotroph axis controls liver damage in NAFLD

The progression from hepatosteatosis to NASH is associated with increased hepatocyte apoptosis and liver damage, which initiate inflammation to clear out dead cells and damaged tissue and to facilitate tissue repair.^34,35^ Increased hepatocyte proliferation is one such attempt to repair liver damage and restore loss of mass,^36–38^ while hepatic stellate cells are also activated and transdifferentiate into myofibroblasts, which produce an excessive amount of extracellular matrix proteins that form fibrous connective tissues to replace normal parenchymal tissues.^34^ Hepatic fibrosis, the wound-healing process mediated by hepatic stellate cells, is a key feature used to determine the severity of NASH. Suppression of the somatotroph axis in response to ER stress associated with NASH suggests that this branch of the ISR might activate the cellular protective program and prevent cell death, resulting in reduced inflammation but compromised parenchymal repair due to repressed hepatocyte proliferation and compensatory fibrosis.

**Figure 4.**
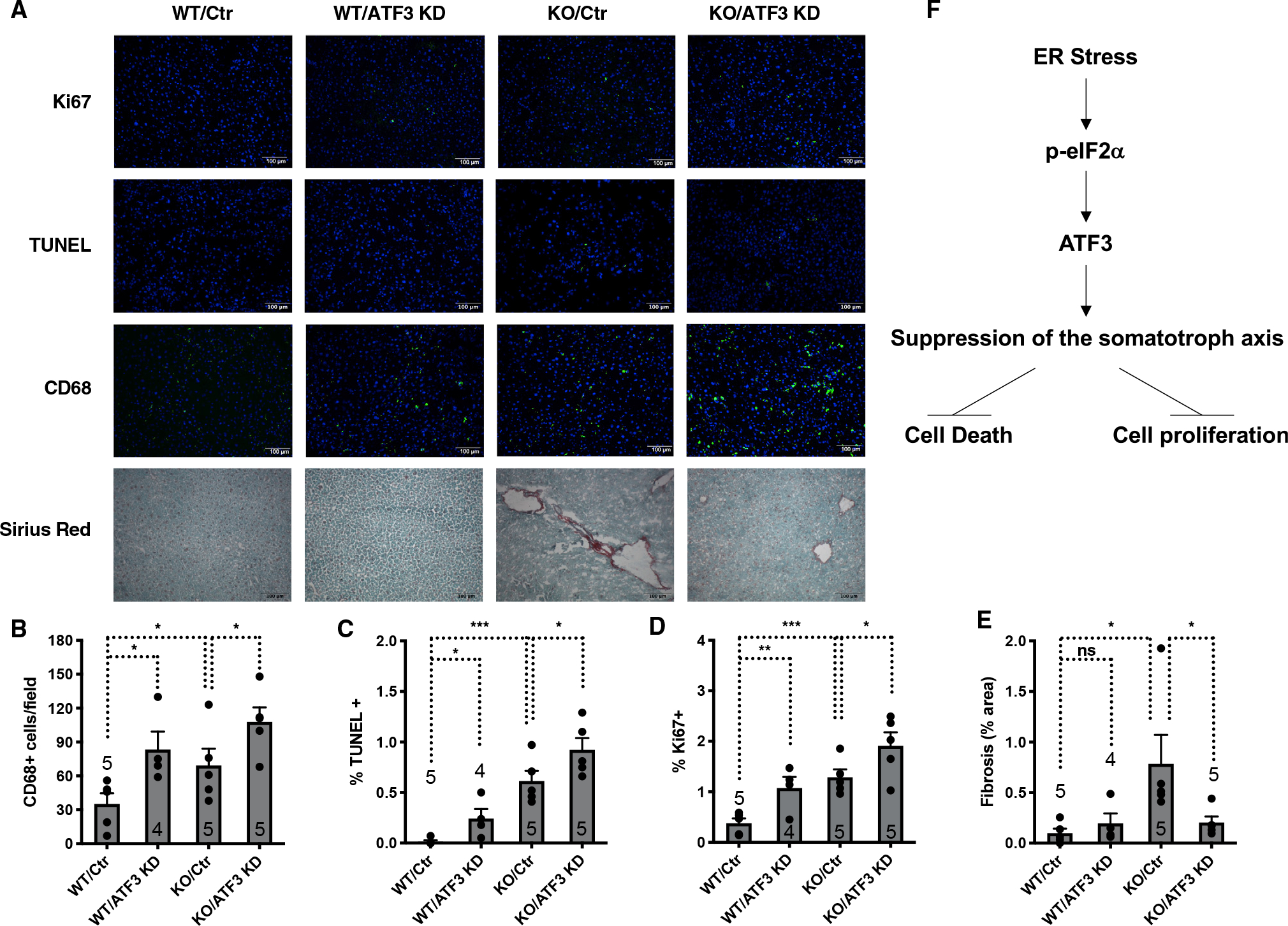
Suppression of the somatotroph axis controls liver damage in NAFLD (A–E) Liver sections stained for Ki67, TUNEL, CD68, and Sirius red (A) and their quantifications (B–E) for *SIRT7*^−/−^ mice and wild-type mice with or without ATF3 knockdown mediated by AAV8-mediated gene delivery. Mice were analyzed 4 weeks after viral infection. n = 4–5 mice. Scale bar: 100 μm. (F) A proposed model. Hepatic ER stress and the ISR induce ATF3 expression and the suppression of the somatotroph axis, leading to reduced hepatocyte death, liver damage, and inflammation, while reducing hepatocyte proliferation and parenchymal repair, resulting in compensatory fibrosis. Error bars represent standard errors. *p < 0.05; **p < 0.01; ***p < 0.001; ns p > 0.05. See also Figure S5.

To test this possibility, we examined the physiological effects of suppressing the somatotroph axis on liver damage in NASH. The livers of *SIRT7*^−/−^ mice exhibited increased inflammation (Figures 4A and 4B), apoptosis (Figures 4A and 4C), proliferation (Figures 4A and 4D), and fibrosis (Figures 4A and 4E), characteristic of the cellular and pathophysiological features of NASH.^21,34–37^ The analysis of the single-cell RNA sequencing data for the livers of wild-type and *SIRT7*^−/−^ mice revealed increased expression of cell-cycle genes in hepatocytes of *SIRT7*^−/−^ mice, consistent with increased proliferation of hepatocytes as a way to repair damage and restore loss of mass (Figure S5). ATF3 inactivation in the livers of *SIRT7*^−/−^ mice via AAV8-mediated gene transfer rescued the suppression of the somatotroph axis (Figure 3K–3P). Liver terminal deoxynucleotidyl transferase-mediated deoxyuridine triphosphate nick end labeling (TUNEL) staining demonstrated increased frequency of apoptotic cells (Figures 4A and 4C), while liver Ki67 staining showed increased frequency of proliferating cells (Figures 4A and 4D) in *SIRT7*^−/−^ mice with ATF3 inactivation compared with *SIRT7*^−/−^ control mice. Compared with *SIRT7*^−/−^ control mice, *SIRT7*^−/−^ mice with ATF3 inactivation showed increased inflammation in the livers as evidenced by staining of CD68, a marker for macrophages (Figures 4A and 4B). Hepatic fibrosis as measured with Sirius red staining was reduced in *SIRT7*^−/−^ mice with ATF3 inactivation (Figures 4A and 4E). Consistent with these observations, ATF3 KO mice showed increased hepatic apoptosis, liver damage, and inflammation upon liver ischemia/reperfusion injury.^39^ These data suggest that suppression of the somatotroph axis prevents hepatocyte apoptosis, liver damage, and inflammation while suppressing hepatocyte proliferation and parenchymal repair and promoting compensatory fibrosis (Figure 4F).

**Figure 5.**
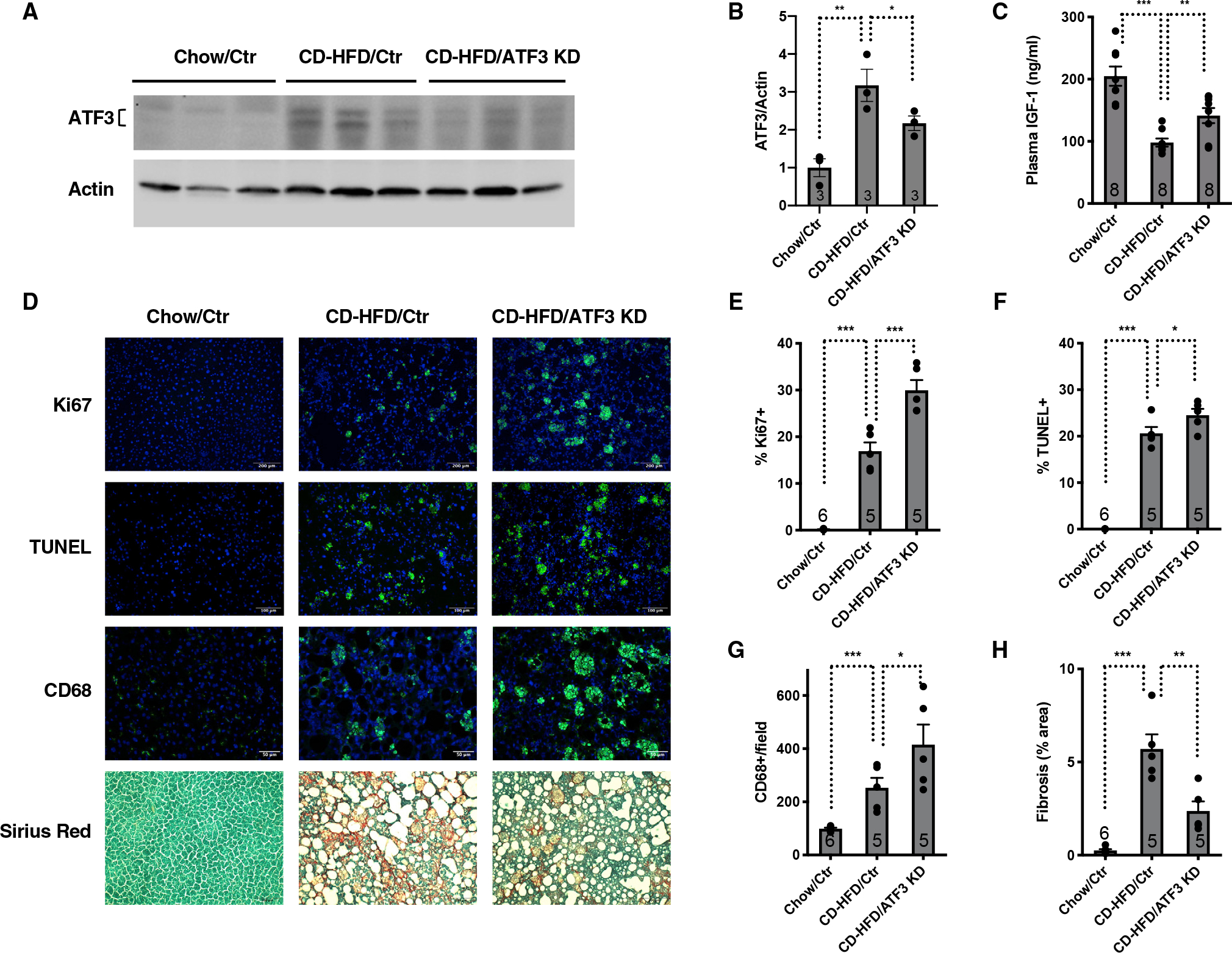
Suppression of the somatotroph axis controls liver damage in mice fed a CD-HFD Comparison of wild-type mice with or without ATF3 knockdown in the livers fed a chow diet or a CD-HFD for 8 weeks. (A and B) Western analyses (A) and quantification (B) of ATF3 in the livers. n = 3 mice. (C) ELISA analyses of plasma levels of IGF-1. n = 8 mice. (D–H) Liver sections stained for Ki67, TUNEL, CD68, and Sirius red (D) and their quantifications (E–H). n = 5–6 mice. Scale bars: 200 (Ki67), 100 (TUNEL, Sirius red), and 50 μm (CD68). Error bars represent standard errors. *p < 0.05; **p < 0.01; ***p < 0.001. See also Figure S6.

Diet-induced NASH mouse models show reduced plasma IGF-1 levels.^40–42^ We therefore next tested whether hepatic ER stress and the ISR suppress the somatotroph axis to control liver damage in commonly used preclinical NASH models. Wild-type mice with ATF3 inactivation in the livers via AAV8-mediated gene transfer and mice treated with control virus were fed a choline-deficient high-fat diet (CD-HFD) to induce hepatic steatosis, liver damage, and fibrosis^35^ (Figure 5A, 5B, S6A, and S6B). ATF3 was induced in the livers of mice fed a CD-HFD compared with mice fed a chow diet (Figures 5A and 5B). Compared with chow-fed mice, CD-HFD mice had reduced expression of the somatotroph genes in the livers (Figures S6C and S6D) and reduced plasma IGF-1 levels (Figure 5C). ATF3 inactivation in the livers of CD-HFD-fed mice increased the plasma IGF-1 levels (Figure 5C). Staining of liver samples showed increased frequency of Ki67 (Figures 5D and 5E), TUNEL (Figures 5D and 5F), and CD68-positive cells (Figures 5D and 5G) and decreased staining of Sirius red (Figures 5D and 5H) in CD-HFD mice with ATF3 inactivation compared with CD-HFD control mice. ATF3 inactivation also increased the expression of inflammatory marker genes in the livers of CD-HFD mice (Figures S6E and S6F).

**Figure 6.**
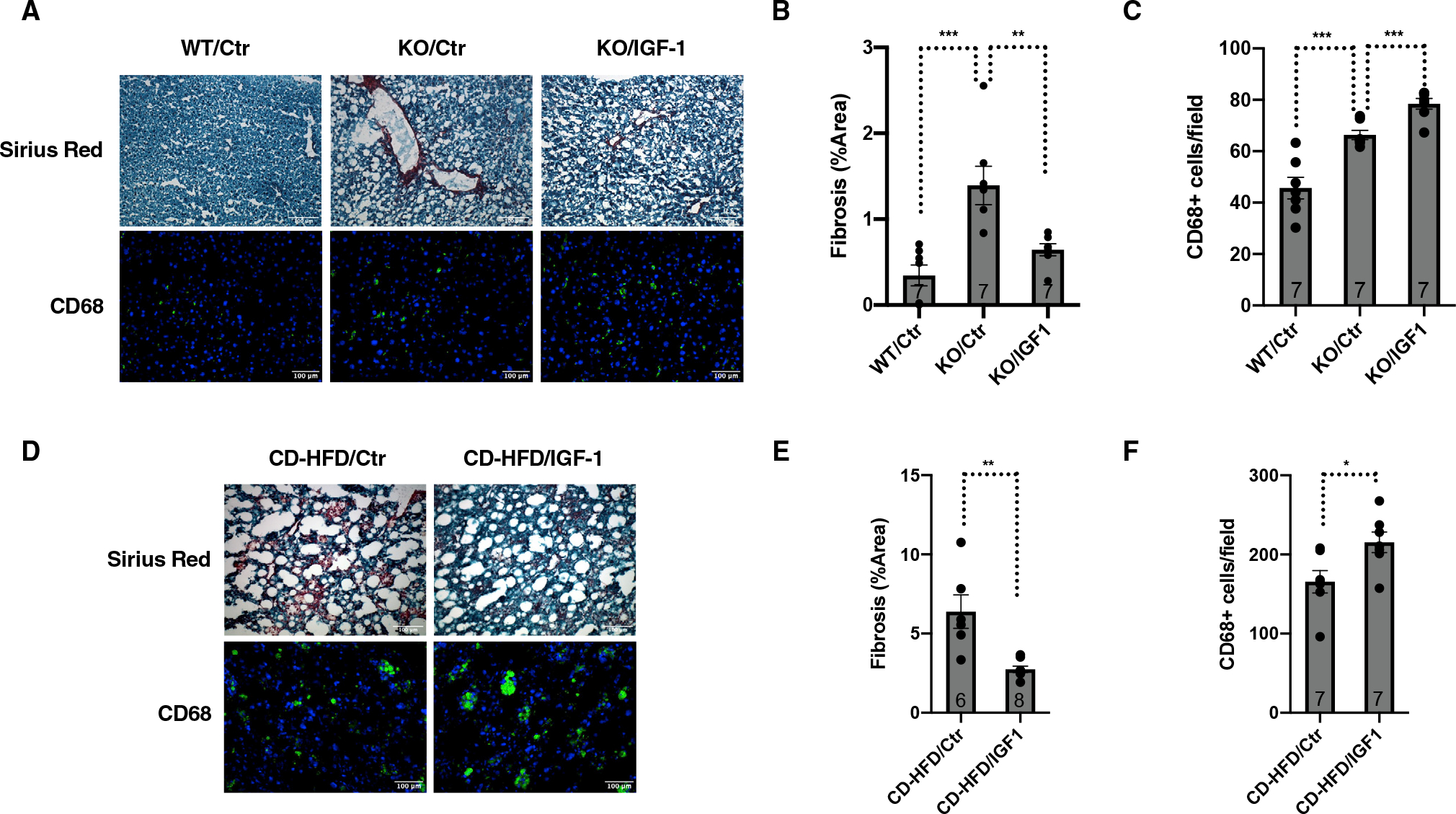
IGF-1 controls liver damage in NAFLD (A–C) Comparison of wild-type and *SIRT7*^−/−^ mice treated with or without IGF-1 for 4 weeks. Data shown are liver sections stained for CD68 and Sirius red (A) and their quantifications (B and C). n = 7 mice. Scale bar: 100 μm. (D–F) Comparison of wild-type mice fed a CD-HFD for 3 weeks followed by treatment with or without IGF-1 for 4 weeks. Data shown are liver sections stained for CD68 and Sirius red (D) and their quantifications (E and F). n = 6–8 mice (E) and 7 mice (F). Scale bar: 100 μm. Error bars represent standard errors. *p < 0.05; **p < 0.01; ***p < 0.001.

To test directly the effects of IGF-1 on liver damage in NASH, we treated either *SIRT7*^−/−^ mice or CD-HFD mice with IGF-1 for 4 weeks. Staining of liver samples showed increased frequency of CD68-positive cells and decreased staining of Sirius red in *SIRT7*^−/−^ mice (Figures 6A–6C) or CD-HFD mice (Figures 6D–6F) treated with IGF-1 compared with their respective controls. These results are consistent with the effects of upregulating the somatotroph axis via ATF3 KD on liver damage in NASH (Figures 4 and 5).

Together, these data are consistent with the model that ATF3 activation represses the somatotroph axis, leading to reduced hepatic apoptosis and inflammation but decreased hepatic proliferation and increased fibrosis (Figure 4F). Therefore, an effective approach to ameliorate both inflammation and fibrosis, two major indications for effective NAFLD therapeutics, would be targeting an event upstream of the suppression of the somatotroph axis, such as ER stress.

### NAD^+^ repletion reduces hepatic ER stress and ameliorates liver damage in NAFLD

**Figure 7.**
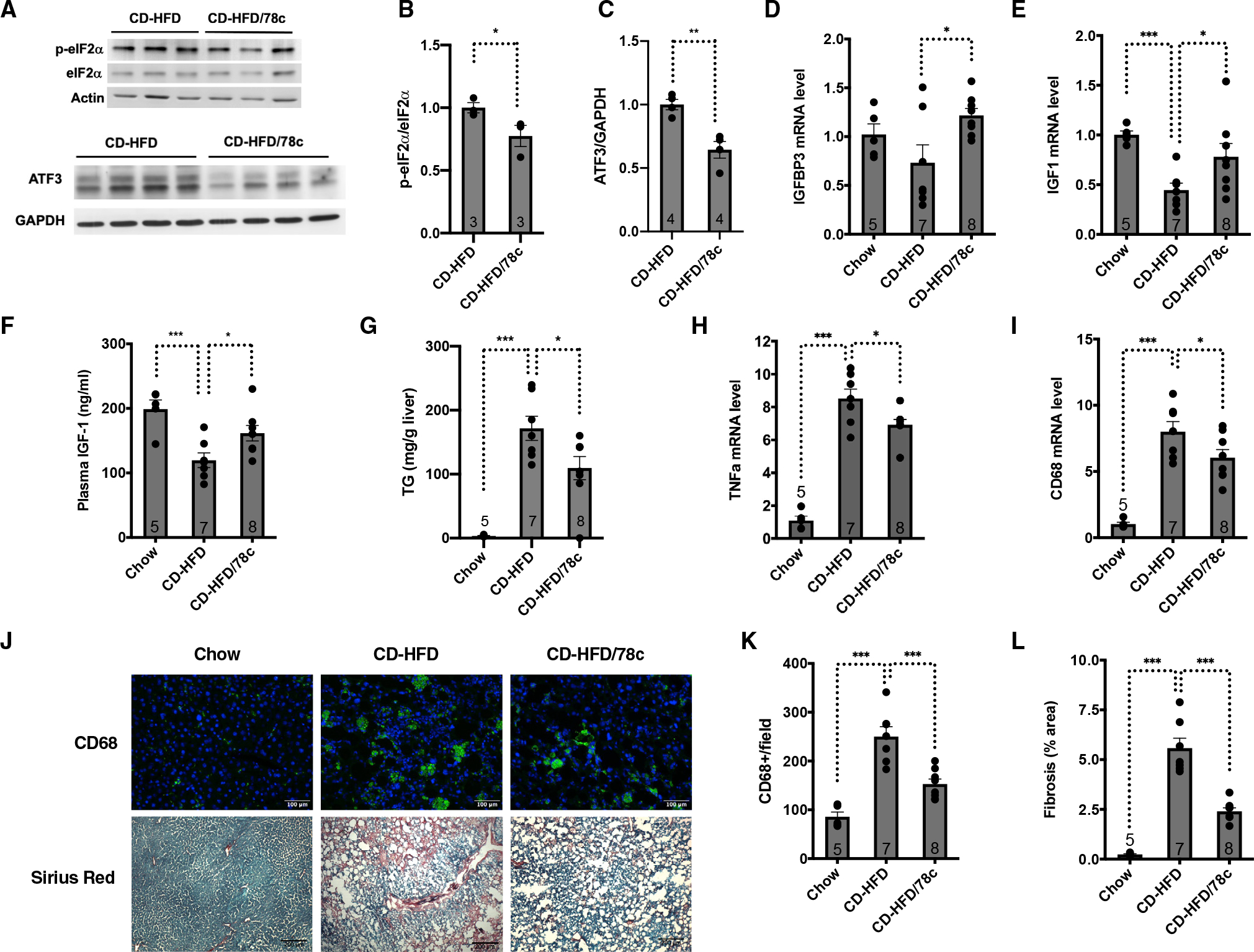
NAD^+^ repletion ameliorates hepatic ER stress, dysregulated somatotroph axis, and liver damage in NAFLD Comparison of mice fed a chow diet or a CD-HFD for 3 weeks followed by treatment with or without 78c for 4 weeks. (A–C) Western analyses (A) and quantification (B and C) for phosphorylated eIF2α and ATF3 in the livers. n = 3–4 mice. (D and E) Quantitative real-time PCR analyses for the mRNA levels of indicated genes in the livers. GAPDH was used as an internal control. n = 5–8 mice. (F) ELISA analyses of plasma levels of IGF-1. n = 5–8 mice. (G) Liver triglyceride quantification. n = 5–8 mice. (H and I) Quantitative real-time PCR analyses for the mRNA levels of the indicated genes in the livers. GAPDH was used as an internal control. n = 5–8 mice. (J–L) Liver sections stained for CD68 and Sirius red (J) and their quantifications (K and L). n = 5–8 mice. Scale bars: 100 (CD68) and 200 μm (Sirius red). Error bars represent standard errors. *p < 0.05; **p < 0.01; ***p < 0.001. See also Figure S7.

We took a pharmacological approach to activate SIRT7 and suppress ER stress. NAD^+^ boosters are emerging to be attractive means to activate sirtuins.^43–45^ We treated CD-HFD mice with 78c, an NAD^+^ booster, for 4 weeks^46^ (Figures S7A and S7B). 78c treatment reduced ER stress and the ISR induction in the liver (Figures 7A–7C), rescued dysregulated somatotroph gene expression (Figures 7D and 7E), increased the plasma IGF-1 levels (Figure 7F), reduced hepatic triglyceride content (Figure 7G), and reduced hepatic inflammation (Figures 7H–7K) and fibrosis (Figures 7J and 7L).

## DISCUSSION

Our studies establish suppression of the somatotroph axis as a physiological response to hepatic ER stress that controls liver damage during the progression of NASH. Suppression of the somatotroph axis results in improved hepatocyte survival and reduced inflammation, but repressed hepatocyte proliferation and parenchymal repair, and compensatory fibrosis (Figures 4, 5, and 6). These findings provide mechanistic insights into the epidemiological observations that suppression of the somatotroph axis is associated with patients with NAFLD, in particular the severity of fibrosis.^10–19^ These findings also offer an explanation that NAFLD can be ameliorated by calorie restriction at the early stage, which elicits the suppressed somatotroph axis and prevents hepatocyte cell death and further liver damage.^1–9^

Our studies identify a regulatory branch of the hepatic ISR and uncover ATF3 as a stress-induced transcription factor that orchestrates the gene expression of the somatotroph axis. Although ATF3 is known to be induced by ER stress,^30^ its role in stress response is obscure. We show that ATF3 binds to the promoters or enhances of the somatotroph genes to control their expression (Figures S4C–S4F and 3A–3F). The suppressed somatotroph axis leads to reduced cell proliferation but increased stress resistance to improve cell survival (Figures 3G–3I). Thus, this regulatory branch of ISR constitutes a stress response to prevent cell death.

Overnutrition and obesity are strongly associated with NAFLD, while calorie restriction is an effective intervention that prevents NAFLD in humans.^20,21,47–50^ Sirtuins are nutrient sensors that mediate the responses to calorie restriction and overnutrition.^26,27,51–55^ Indeed, evidence is emerging showing dysregulated sirtuin expression in the livers of patients with NAFLD^56^ and linking sirtuins to nutritional regulation of PNPLA3, which is strongly linked to NAFLD.^57^ SIRT7 alleviates diet-induced NAFLD.^27^ Therefore, sirtuins are thought to be relevant to the pathogenesis and prevention of NAFLD associated with nutrition and obesity.

Furthermore, dysregulated NAD^+^ metabolism has been linked to human NAFLD. For example, the levels of NAMPT, a rate-limiting enzyme for NAD^+^ biosynthesis, is reduced in the livers and plasma of patients with NAFLD.^58^ NAMPT functions to prevent hepatocyte apoptosis.^58^ The NAD^+^ level is reduced in the livers of patients with NASH.^59^ Sirtuins are the major NAD^+^-consuming enzymes that mediate the signaling effects of NAD^+^ and are thought to be the mediators of NAD^+^ metabolism in NAFLD. Indeed, overexpression of SIRT7 rescues diet-induced NAFLD in mice.^27^

Given the association of sirtuins to known risk factors of NAFLD, such as diet, obesity, and NAD^+^, the prominent NAFLD phenotype in the *SIRT7*^−/−^ mouse model,^26–28^ and the observation that SIRT7 prevents the development of NAFLD by suppressing ER stress,^27^ a major driver of the progression from NAFLD to NASH,^22^ the *SIRT7*^−/−^ mouse model is relevant to human NASH, although human genome-wide association study (GWAS) data linking SIRT7 to NAFLD have not emerged yet. Indeed, our single-cell RNA sequencing analysis provided further support that the *SIRT7*^−/−^ mouse model develops NAFLD (Figures 1B, 1C, S1D, and S1E). Using the *SIRT7*^−/−^ mouse model, we showed that suppression of the somatotroph axis reduces hepatic inflammation but promotes fibrosis (Figures 4 and 6A–6C). This finding was further validated using the CD-HFD mouse model (Figure 5, 6D–6F, 7, S6, and S7). The consistent findings in both mouse models of NAFLD further support the relevance of the *SIRT7*^−/−^ mouse model to NAFLD.

NAD^+^ boosting has demonstrated therapeutic potential for a number of diseases.^43–45^ Our studies show that NAD^+^ boosting via 78c can ameliorate NASH, a prevalent metabolic disease that needs a cure, at least in part by modulating the hepatic ISR and the somatotroph axis in mouse models (Figure 7), demonstrating the therapeutic potential of modulating this pathway. Suppression of the somatotroph axis in response to ER stress uncouples inflammation and fibrosis (Figures 4, 5, and 6), providing a basis for combination therapies or targeting an initiating event, such as ER stress, for this metabolic disease (Figure 7).

### Limitations of the study

The role of the hepatic ISR and the somatotroph axis in controlling liver damage during NAFLD has been tested using two NAFLD mouse models, *SIRT7*^−/−^ mice and CD-HFD mice. How this pathway operates in other NAFLD models has not been tested. The effects of IGF1 treatment were examined in mice treated for 4 weeks, but the effects after longer or shorter treatments have not been tested.

## STAR★METHODS

### RESOURCE AVAILABILITY

#### Lead contact

Further information and requests for resources and reagents should be directed to and will be fulfilled by the lead contact, Danica Chen (danicac@berkeley.edu).

#### Materials availability

Unique reagents generated in this study are available from the lead contact, Danica Chen (danicac@berkeley.edu).

#### Data and code availability

The sequencing data reported in this paper has been deposited in NCBI′ s Gene Expression Omnibus and are accessible through GEO: GSE216996. This paper does not report custom code.

Any additional information required to reanalyze the data reported in this paper is available from the lead contact upon request (danicac@berkeley.edu).

### EXPERIMENTAL MODEL AND SUBJECT DETAILS

#### Mice

SIRT7^−/−^ mice have been described previously.^27,53^ For a diet-induced NAFLD mouse model, 8-week-old C57BL/6 male mice were fed with choline-deficient high-fat diet (Research Diet, A06071302) consisting of 60 kcal% fat with 0.1% methionine and no added choline for 3 weeks before either 78c treatment or IGF-1 treatment. 78c was administered to mice by intraperitoneal injection (10 mg/kg/dose) twice daily for 4 weeks. Control mice received vehicle (5% DMSO, 15% PEG400, 80% of 15% hydroxypropyl-g-cyclodextrin (in citrate buffer pH 6.0)) injections. IGF-1 (Pepro Tech) dissolved in 0.1% BSA/PBS was administered to mice by subcutaneous injection (20 μg/kg/day) for 4 weeks. For IGF-1-treated SIRT7^−/−^ mice, 7- to 11-month-old mice were used for the experiment. All mice were housed on a 12:12 h light:dark cycle at 25°C and were given free access to food and water. All animal procedures were in accordance with the animal care committee at the University of California, Berkeley.

#### Cell culture

Hepa 1–6 cells were acquired from cell culture facility at the University of California, Berkeley. Cells were cultured in advanced Dulbecco’s modified Eagle’s medium (Gibco) supplemented with 10% FBS (Gibco). For ER stress induction, cells were treated with tunicamycin (Sigma, 2 μg/mL) or thapsigargin (Sigma, 0.1μM) for 24 h before biochemical analysis. For ATF3 knockdown, Hepa 1–6 cells were transfected with AllStars Negative Control siRNA (Qiagen, 1027281) or ATF3 siRNA (Qiagen, GS11910) using RNAiMAX (Invitrogen, 13778100) according to manufacture’s instruction. To generate Hepa 1–6 cells with stable ATF3 knockdown, cells were infected with lentivirus. For lentiviral packaging, 293T cells were co-transfected with packaging vectors (pCMV-dR8.2 dvpr and pCMV-VSV-G) and the pLKO.1-ATF3 shRNA (Sigma, TRCN0000082129, TRCN0000082132) or control construct. Viral supernatant was harvested after 48 h and 72 h after transfection, as described previously.^64^ For transduction, cells were incubated with virus-containing supernatant in the presence of 10 μg/mL polybrene. After 48 h, infected cells were selected with puromycin (4 μg/mL). For cell proliferation, 0.3 × 10^6^ cells were seeded in a 6-well plate. Two days later, 20% cells were passaged to a new well and were counted 24 h later.

Primary hepatocytes were suspended in plating medium (DMEM low glucose, 5% FBS and 1%Pen/Strep) and plated on collagen-coated cell culture plates (Sigma-Aldrich C3867-1VL). After 3 h, it was changed to maintenance media (Williams E media, 1% Glutamine and 1% Pen/Strep). The next day cells were treated with tunicamycin for 24 h (Sigma, 4 μg/mL) before analysis.

### METHOD DETAILS

#### Apoptosis assay

Apoptotic cells were assayed using propidium iodide (BioLegend) and FITC Annexin V staining (BioLegend) according to the manufacturer’s instruction (BioLegend). All data were collected on an LSR Fortessa (BD Bioscience), and data analysis was performed with FlowJo (TreeStar).

#### Chromatin immunoprecipitation

Cells were prepared for ChIP as previously described,^65^ with the exception that DNA was washed and eluted using a QIAprep Spin Miniprep kit (Qiagen) rather than by phenol-chloroform extraction. For ChIP with mouse livers, 150 mg mouse liver were minced and dounce homogenized with 10 strokes in hypotonic lysis buffer (10 mM HEPES, pH7.5, 10 mM KCl, 1.5 mM MgCl2, 250 mM Sucrose, 0.5% NP40, and protease inhibitor cocktail). Lysates were filtered through a 100um cell strainer and spin at 1500 g for 5 min. Lipid and cytoplasmic fractions were removed and the nuclear pellet was resuspended in lysis buffer, cross-linked with fresh formaldehyde (1%) for 5 min at room temperature, quenched with glycine (125 mM), and washed twice with PBS.

#### Affymetrix microarray

Total RNA was isolated from the livers of wild type and SIRT7^−/−^ mice using an RNA isolation kit (Qiagen). Microarray hybridizations were performed at the University of California, Berkeley Functional Genomics Laboratory using Affymetrix GeneChip mouse 430As according to the instructions of the manufacturer (Affymetrix). RMA normalization was applied and the limma package was used to identify the differentially expressed genes. Differentially expressed genes were selected using the Benjamini-Hochberg method to control the FDR at 15%.

#### Single-cell RNA-sequencing of livers using 10x Genomics Chromium

5- to 6-month-old SIRT7^−/−^ mice were used for single-cell RNA-sequencing of livers. Hepatocytes and non-parenchymal cells (NPCs) were isolated by a two-step collagenase perfusion method.^66^ Briefly, after the inferior vena cava was cannulated with a 25 gauge catheter and the portal vein was cut, the liver was perfused at 10 mL/min with Liver Perfusion Medium (Gibco 17701-038) at 37°C for 5 min, followed by perfusion with collagenase type IV (Worthington LS004188) in HBSS (GIBCO) at 37°C for 5 min. The liver was dissected out and transferred to Petri dish with William E medium (Gibco 12551-032) containing 200 mM L-glutamine, 1% pen/strep and 1% non-essential amino acid. Then gently shake out the cells from liver capsule. The released liver cells were passed through a 100 μm filter. Hepatocytes were separated from NPCs by low-speed centrifugation (50 × g, 4 min, 3x, brake = 2) and further purified by Percoll gradient centrifugation (50% v/v) to remove dead cells.^67^ NPCs were pelleted from supernatant by centrifugation (300 xg, 10 min) then purified by Percoll gradient centrifugation (33% v/v) to remove dead cells.^68^ Cell viability was confirmed by trypan blue exclusion. 3,000 hepatocytes and 3,000 NPCs were mixed and used directly for scRNA-seq analysis using 10X Genomics Chromium Single-Cell 3′ according to the manufacturer’s instructions.

#### 10x genomics single-cell RNA-sequencing data pre-processing, UMAP analysis, and identification of cell clusters

RNA reads from sequencing were demultiplexed and aligned to mouse transcriptome (mm10) using the Cell Ranger software (10x Genomics, v.6.0.0). The Scanpy Python package (v.1.6.0) was used for the pre-processing of the single-cell RNA seq data.^60^ Cells with less than 500 unique genes or more than 5% mitochondrial genes were removed. Genes detected in less than 3 cells were excluded. We included 11,610 cells with 3,270 cells from wild type and 8,340 cells from SIRT7^−/−^, and 16,623 genes for further analysis. The data was normalized such that every cell has 10,000 counts and then log transformed with an offset of 1. The batch correction was done by the bbknn batch-alignment algorithm.^61^ We computed the highly variable genes with the top 1,000 genes and the flavor set to ‘cell_ranger’. The highly variable genes were used for principal components analysis. The data was visualized by UMAP (Uniform Manifold Approximation and Projection) projection using Scanpy. Unsupervised clustering was done by the Leiden algorithm^62^ with a resolution of 0.35. Marker genes for each cluster were calculated by Wilcoxon rank-sum test. The cell identity of each cluster was determined by comparing the marker genes of each cluster with the marker genes identified in the literature.

#### Differential gene expression analysis, bar plots, violin plots, and dot plots for gene expression in single cells, and pathway enrichment analysis

Adaptive thresholding of the single-cell gene expression data was performed with the MAST R package (v1.12.0), and differential gene expression analysis of wild type and SIRT7^−/−^ cells from each cluster using a hurdle model with the wild type cells as the ref. 63. To visualize the expression of genes, log-normalized expressions of genes were extracted from the data after adaptive thresholding and plotted for every cell with a violin plot and an overlying strip plot by the Seaborn Python package (v.0.9.0). The bar plots were generated by Seaborn. The UMAP plots, dot plots, and track plots were generated by Scanpy. The GSEAPY Python package (v.0.10.3) was used for pathway enrichment analysis.

#### Quantitative real-time PCR

RNA was isolated from cells or tissues using Trizol reagent (Invitrogen) following the manufacturer’s instructions. cDNA was generated using the qScript cDNA SuperMix (Quanta Biosciences). Gene expression was determined by quantitative real-time PCR using Eva qPCR SuperMix kit (BioChain Institute) on an ABI StepOnePlus system. All data were normalized to GAPDH expression.

#### AAV8-mediated gene transfer

For AAV8-mediated gene transfer to the mouse liver, Myc knockdown target sequence was cloned into dsAAV-RSVeGFP-U6 vector. AAV8 for knocking down Myc was produced by Vigene biosciences. AAV8 for knocking down ATF3 was acquired from Vector biolabs. Myc knockdown target sequence: 5′-CCCAAGGTAGTGATCCTCAAA-3′. ATF3 knockdown target sequence: 5′-TGCTGCCA AGTGTCGAAACAA-3′. Each mouse was injected with 3 × 10^11^ genome copies of virus via tail vein. Mice were characterized four weeks after viral infection (5- to 6-month-old wild-type and SIRT7^−/−^ mice) or eight weeks after viral infection (8-week-old C57BL/6 mice on CD-HFD).

#### Plasma IGF-1 levels

To detect IGF-1 in the plasma, the plasma was pretreated with acid-ethanol extraction solution to release IGF-1 from binding proteins. Briefly, 120 μL of acid-ethanol extraction buffer (hydrochloric acid:water:ethanol = 1:4:35, v/v/v) was added to 30 μL of plasma. The extract was incubated for 30 min at room temperature with shaking. The extract was centrifuged at 10,000 rpm for 5 min and 100 μL of supernatant was collected. 200 uL of Tris buffer (pH = 7.6) was added to the supernatant. IGF-1 was detected using IGF-1 Mouse ELISA Kit (Invitrogen).

#### Immunohistochemistry

Tissue sections (5 μm) were mounted on glass slides. Slides were fixed with 10% formalin. Tissue processing and immunohistochemistry was performed on sections. Primary antibodies were: mouse anti-CD68 (Biolegend, 137001); Ki67 (Biolegend, 652409). After overnight incubation, primary antibody staining was revealed using fluorescence conjugated secondary antibodies. Nuclei were counter stained using DAPI. Images were taken with Zeiss AxioImager microscope. The positive cells were manually counted or counted using ImageJ.

#### Fibrosis staining

Liver sections were fixed with 10% formalin and then stained with Sirius Red (Sigma)/Fast Green (Sigma). Images were taken with Zeiss AxioImager microscope. The positive area was quantified using ImageJ.

#### TUNEL staining

Apoptosis was detected with Apo-Brdu *in situ* DNA fragmentation assay kit according to the manufacturer’s instruction (Biovision). Nuclei were counter stained using DAPI. TUNEL-positive cells were imaged using Zeiss AxioImager microscope.

#### Western blot

Tissues or cells were homogenized in a lysis buffer that contained protease inhibitor, and total protein was extracted with gentle rotation for 30 min at 4°C. The extract was centrifuged at 15,000 g for 15 min at 4°C. Supernatants were collected and total protein was quantified with BCA assay (Thermo Scientific, 23225). Proteins were resolved by SDS-PAGE and transferred to nitrocellulose membranes (Bio-Rad), which was incubated with specific primary antibodies and horseradish peroxidase-conjugated secondary antibodies, and enhanced chemiluminescence substrate (PerkinElmer, NEL103001EA), and visualized using ImageQuant^™^ LAS 4000 (GE Healthcare).

#### Triglyceride quantification

Triglycerides were extracted from liver tissues as described.^69^ Briefly, liver tissues were homogenized in the methanol/chloroform buffer (1:2, v/v) and lipids were extracted with gentle rotation for 2 h at room temperature. The homogenate was centrifuged at 15,000 g for 5 min. Supernatants were concentrated via nitrogen gas and reconstituted with the reconstitution buffer (1% Triton X-100 in 100% ethanol). Extracted triglyceride was quantified in accordance with the manufacturer’s instruction (Wako Diagnostics).

### QUANTIFICATION AND STATISTICAL ANALYSIS

Mice were randomized to groups and analysis of mice and tissue samples was performed by investigators blinded to the treatment or the genetic background of the animals during experiments. Statistical analysis was performed with Student’s t test (Excel) unless specified. Wilcoxon rank-sum test for single-cell RNA sequencing analysis was performed using the SciPy Python package (v.1.4.1). Data are presented as means and error bars represent standard errors. In all corresponding figures, * represents p < 0.05. ** represents p < 0.01. *** represents p < 0.001. ns represents p > 0.05. Replicate information is indicated in the figures.

## Supplementary Material

1

2

3

4

5

## Figures and Tables

**KEY RESOURCES TABLE:** 

REAGENT or RESOURCE	SOURCE	IDENTIFIER

Antibodies		

p-eIF2α (Ser52) polyclonal antibody	Invitrogen	Cat# 44728G; RRID:AB_1500038
eIF2α antibody	CST	Cat# 9722; RRID: AB_2230924
Phospho-Akt (Ser473) antibody	CST	Cat# 9271; RRID:AB_329825
Akt antibody	CST	Cat# 9272; RRID:AB_329827
Actin antibody	Sigma	Cat# A2066; RRID:AB_476693
GAPDH antibody	CST	Cat# 5174; RRID: AB_10622025
Mouse growth hormone R/GHR antibody	R&D	Cat# AF1360; RRID:AB_2111403
Mouse FGF acidic/FGF1 antibody	R&D	Cat# AF4686; RRID: AB_2924726
ATF-3 (D2Y5W) Rabbit antibody	CST	Cat# 33593S; RRID: AB_2799039
Normal Rabbit IgG	CST	Cat# 2729S; RRID: AB_1031062
Purified anti-mouse CD68 antibody	BioLegend	Cat#137001; RRID: AB_2044003
Goat anti-rat IgG (H + L) cross-absorbed secondary antibody, DyLight 488	ThermoFisher Scientific	Cat# SA5-10018; RRID: AB_2556598
FITC anti-mouse Ki-67 antibody	BioLegend	Cat# 652409; RRID: AB_2562140

Chemicals, peptides, and recombinant proteins		

78c (CD38 inhibitor)	MedChemExpress	Cat# HY-123999; CAS#1700637-55-3
Dimethyl Sulfoxide (DMSO)	Sigma	Cat# D8418
Polyethylene glycol 400 (PEG400)	Sigma	Cat# PX1286B
Hydroxypropyl-g-cyclodextrin	Santa Cruz biotechnology	Cat# sc-238090A
Recombinant human IGF1	PeproTech	Cat# 100-11
BSA	Sigma	Cat# A7906
Dulbecco’s Modification of Eagle’s medium	Gibco	Cat# 11995065
Dulbecco’s Modification of Eagle’s medium (low glucose)	Gibco	Cat# 11885-084
Williams E media	Gibco	Cat# 12551-032
Liver perfusion medium	Gibco	Cat# 17701-038
Collagenase type IV	Worthington	Cat# LS004188
L-Glutamine	Gibco	Cat# 25030081
Non-essential amino acid (100X)	Gibco	Cat# 11140-050
Percoll^™^ PLSU	Cytiva	Cat# 17544702
Fetal Bovine Serum	Invitrogen	Cat#10437-028
Tunicamycin	Sigma	Cat# T7765
Thapsigargin	Sigma	Cat# T9033
RNAiMAX	Invitrogen	Cat# 13778100
Sirius red (direct red 80)	Sigma	Cat# 365548
Fast green	Fisher Chemical	Cat# F99-10
qScript^™^ cDNA SuperMix	Quanta biosciences	Cat# 95048
qPCR SuperMix kit	BioChain Institute	Cat# K5052400
Penicillin Streptomycin solution (100x)	Invitrogen	Cat# 15140122
Collagen, type I solution from rat tail	Sigma	Cat# C3867-1VL
Trypsin-EDTA (0.25%)	Gibco	Cat# 25200056
TRIzol reagent	Invitrogen	Cat# 15596026
Lipofectamine 2000	Invitrogen	Cat# 11668019
HEPES	Gibco	Cat# 15630080
HBSS, calcium, magnesium, no phenol red	Gibco	Cat# 14025092
HBSS, no calcium, no magnesium, no phenol red	Gibco	Cat# 14175095
Western (blotting) Lightning Plus-ECL substrate	Perkin Elmer	Cat# NEL103E001EA
DAPI (4′,6-diamidino-2-phenylindole, dihydrochloride)	Thermo Fisher Scientific	Cat#62247
Propidium iodide solution	Biolegend	Cat#421301
FITC Annexin V	BioLegend	Cat# 640906
Formaldehyde	Thermo Fisher Scientific	Cat# F79-500

Critical commercial assays		

QIAprep spin Miniprep kit	Qiagen	Cat# 27106X4
10× Genomics single Cell 3′ reagent kits v3	10× Genomics	Cat# PN-1000075
IGF-1 mouse ELISA kit	Invitrogen	Cat# EMIGF1
Apo-Brdu *in situ* DNA fragmentation assay kit	Biovision	Cat# K401
Pierce^™^ BCA protein assay kit	Thermo Scientific	Cat# 23225
L-type Triglyceride M enzyme color A	Fujifilm Wako Diagnostics	Cat# 996-02895
L-type Triglyceride M enzyme color B	Fujifilm Wako Diagnostics	Cat# 992-02995

Deposited data		

SIRT7 liver		GEO: GSE216996

Experimental models: Cell lines		

Hepa 1–6	UC Berkeley Cell culture facility	N/A
HEK293T	ATCC	CRL-3216

Experimental models: Organisms/strains		

Mouse: SIRT7 KO	Shin et al.^27^	N/A
Mouse: C57BL/6J	National Institute on Aging	N/A

Oligonucleotides		

qPCR primer sequences	IDT (integrated DNA technologies)	Table S2
IGFBP3 ChIP Forward primer: GTTCTCGCTGGGAAATGCCT	IDT (integrated DNA technologies)	N/A
IGFBP3 ChIP Reverse primer: TCAGCGCCTGTGTACTTTGT	IDT (integrated DNA technologies)	N/A
IGF-1R ChIP Forward primer: GGGAATTTCGTCCCAAATAAAAGGA	IDT (integrated DNA technologies)	N/A
IGF-1R ChIP Reverse primer: GAGAGAAACACGAGCCCCC	IDT (integrated DNA technologies)	N/A
Tubulin ChIP Forward primer: AGACGGAAGAGAACACTGCG	IDT (integrated DNA technologies)	N/A
Tubulin ChIP Reverse primer: CTTCATCGGGCTTCAGTCGT	IDT (integrated DNA technologies)	N/A
ATF3 siRNA TGCTGCCAAGTGTCGAAACAA	Qiagen	Cat# GS11910
Control siRNA	Qiagen	Cat# 1027281
Myc siRNA CCCAAGGTAGTGATCCTCAAA	Shin et al.^27^	N/A

Recombinant DNA		

pCMV-dR8.2 dvpr	Addgene	Plasmid: #8455
pCMV-VSV-G	Addgene	Plasmid: #8454
pLKO.1-ATF3	Sigma	TRCN0000082129 TRCN0000082132
dsAAV-RSVeGFP-U6	Shin et al.^27^	N/A
dsAAV-RSVShMyc	Shin et al.^27^	N/A
Ad-m-ATF3-shRNA	Vector biolabs	Cat# shADV-253206

Software and algorithms		

Cell ranger (v.6.0.0)	10X Genomics	N/A
Scanpy Python package (v.1.6.0)	Luo et al.^60^	https://github.com/scverse/scanpy
Bbknn batch-alignment algorithm	Polański et al.^61^	https://github.com/Teichlab/bbknn
Leiden algorithm	Traag et al.^62^	https://github.com/vtraag/leidenalg
MAST R package (v.1.12.0)	Finak et al.^63^	https://github.com/RGLab/MAST
Seaborn Python package (v.0.9.0)		https://seaborn.pydata.org/citing.html
GSEAPY Python package (v.0.10.3)		https://github.com/zqfang/GSEApy/releases
ImageJ		https://imagej.nih.gov/ij/
iVision (v.4.5.6 r4)	BioVision Technologies	https://www.biovis.com
GraphPad Prism	GraphPad	https://www.graphpad.com/

Other		

Choline-deficient high fat diet	Research Diet	Cat# A06071302

## Highlights

- Hepatic ER stress suppresses the somatotroph axis by inducing ATF3
- Suppression of the somatotroph axis controls liver damage in NAFLD
- NAD^+^ repletion ameliorates dysregulated somatotroph axis and liver damage in NAFLD
