# Supplementary material for: The hepatic integrated stress response suppresses the somatotroph axis to control liver damage in nonalcoholic fatty liver disease
Source: Cell Rep. Author manuscript; Available in PMC 2023 Jan 7. (PMC9825120; doi:10.1016/j.celrep.2022.111803)
Supplement: 1 [file NIHMS1859705-supplement-1.pdf]

Cell Reports, Volume *41*

Supplemental information

The hepatic integrated stress response suppresses the somatotroph axis to control liver damage in nonalcoholic fatty liver disease

Rika Ohkubo, Wei-Chieh Mu, Chih-Ling Wang, Zehan Song, Marine Barthez, Yifei Wang, Nathaniel Mitchener, Rasul Abdullayev, Yeong Rim Lee, Yuze Ma, Megan Curtin, Suraj Srinivasan, Xingjia Zhang, Fanghan Yang, Peter H. Sudmant, Angela Oliveira Pisco, Norma Neff, Cole M. Haynes, and Danica Chen

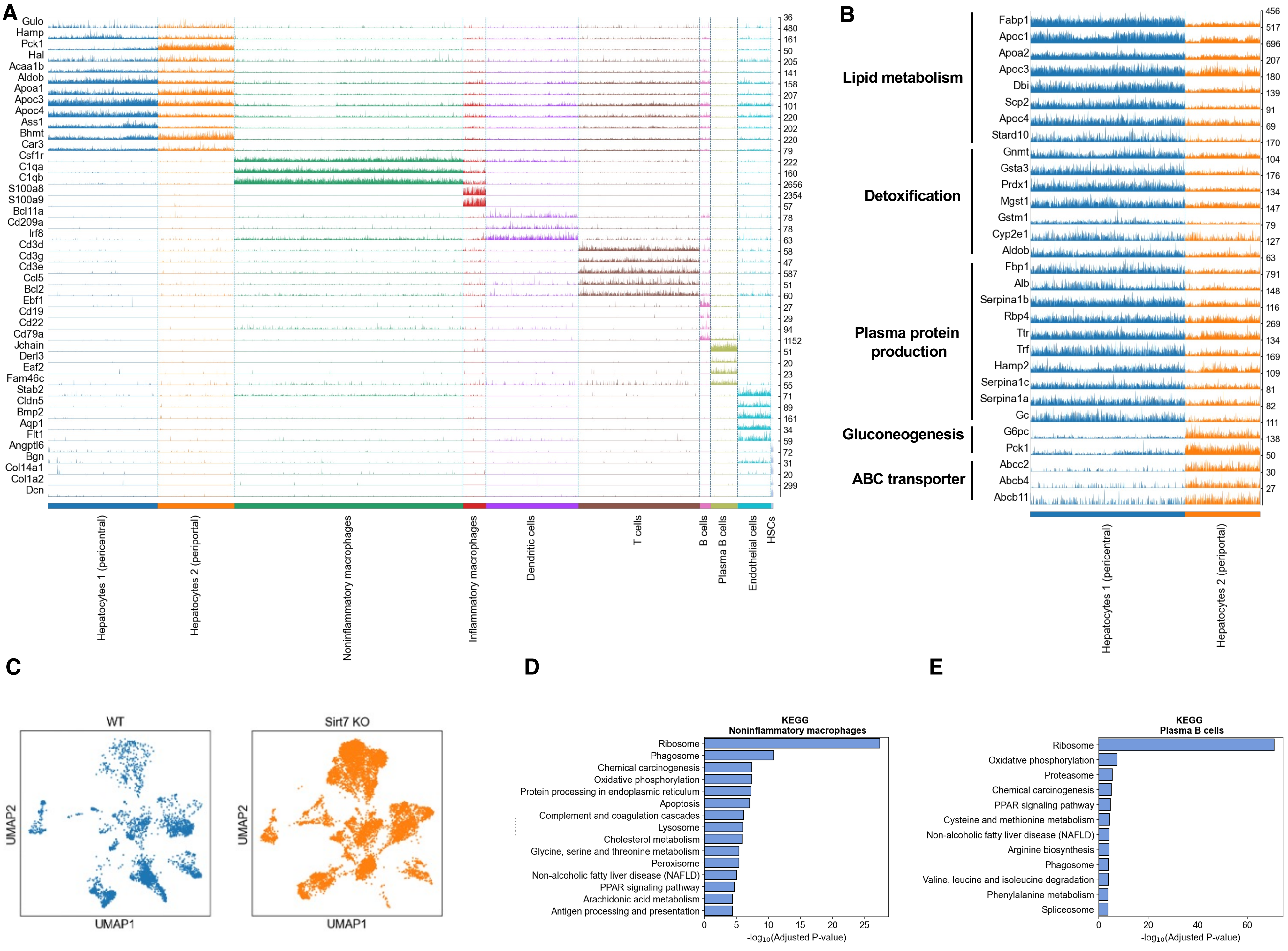
A
Gulo
Hamp
Pck1
Hal
Acaa1b
Aldob
Apoa1
Apoc3
Apoc4
Ass1
Bhmt
Car3
Csf1r
C1qa
C1qb
S100a8
S100a9
Bcl11a
Cd209a
Irf8
Cd3d
Cd3g
Cd3e
Ccl5
Bcl2
Ebf1
Cd19
Cd22
Cd79a
Jchain
Derl3
Eaf2
Fam46c
Stab2
Cldn5
Bmp2
Aqp1
Flt1
Angptl6
Bgn
Col14a1
Col1a2
Dcn
Hepatocytes 1 (pericentral)
Hepatocytes 2 (periportal)
Noninflammatory macrophages
Inflammatory macrophages
Dendritic cells
T cells
B cells
Plasma B cells
Endothelial cells
HSCs
B
Lipid metabolism
Fabp1
Apoc1
Apoa2
Apoc3
Dbi
Scp2
Apoc4
Stard10
Detoxification
Gnmt
Gsta3
Prdx1
Mgst1
Gstm1
Cyp2e1
Aldob
Plasma protein production
Fbp1
Alb
Serpina1b
Rbp4
Ttr
Trf
Hamp2
Serpina1c
Serpina1a
Gc
Gluconeogenesis
G6pc
Pck1
ABC transporter
Abcc2
Abcb4
Abcb11
Hepatocytes 1 (pericentral)
Hepatocytes 2 (periportal)
C
WT
Sirt7 KO
UMAP1
UMAP2
D
KEGG
Noninflammatory macrophages
Ribosome
Phagosome
Chemical carcinogenesis
Oxidative phosphorylation
Protein processing in endoplasmic reticulum
Apoptosis
Complement and coagulation cascades
Lysosome
Cholesterol metabolism
Glycine, serine and threonine metabolism
Peroxisome
Non-alcoholic fatty liver disease (NAFLD)
PPAR signaling pathway
Arachidonic acid metabolism
Antigen processing and presentation
-log10(Adjusted P-value)
E
KEGG
Plasma B cells
Ribosome
Oxidative phosphorylation
Proteasome
Chemical carcinogenesis
PPAR signaling pathway
Cysteine and methionine metabolism
Non-alcoholic fatty liver disease (NAFLD)
Arginine biosynthesis
Phagosome
Valine, leucine and isoleucine degradation
Phenylalanine metabolism
Spliceosome
-log10(Adjusted P-value)

Figure S1. A mouse model of NAFLD. Related to Figure 1.
A, B, Quality control for 10x Genomics single-cell RNA-sequencing data of the livers of wild-type and SIRT7$^{-/-}$ mice. Track plot showing the expression of representative marker genes for each cell cluster. Each bar represents a cell and cells are grouped based on clustering. The cell identity assigned to each cluster is indicated at the bottom. Numbers on the right indicate maximum detected expression. The gene expression is represented by height (y values). Pericentral hepatocytes express highly genes for lipid metabolism, detoxification, and plasma protein production while periportal hepatocytes express highly genes for gluconeogenesis and ABC transporter.
C, Single-cell RNA-sequencing of the livers of WT and SIRT7$^{-/-}$ mice using the 10x Genomics Chromium platform. UMAP clustering of single cell transcriptomes (3270 cells from WT and 8340 cells from SIRT7$^{-/-}$ mice) colored by genotype. n=3 mice. Refer to Figure 1A for cell identity of each cluster.
D, E, Pathway analysis for the biological function of differentially expressed genes in non inflammatory macrophages and plasma B cells of the livers of WT and SIRT7$^{-/-}$ mice.

Gene Title	Gene Symbol	Fold Change	p Value
Growth Hormone Receptor	Ghr	-1.3	0.0019
Fibroblast Growth Factor 1	Fgf1	-1.58	0.0018
Epidermal Growth Factor Receptor	Egfr	-1.39	0.02
Fibroblast Growth Factor Receptor 4	Fgfr4	-1.87	4.16E-06
Prolactin Receptor	Prlr	-2.64	0.0009
IGF Binding Protein, Acid Labile	Igfals	-1.64	0.0003
IGF Binding Protein 3	Igfbp3	-2.16	0.04
IGF Binding Protein 1	Igfbp1	3.74	0.0048
IGF Binding Protein 7	Igfbp7	1.28	0.005
IGF Binding Protein 6	Igfbp6	1.19	0.04

Figure S2. Suppressed somatotroph gene expression in the livers of SIRT7$^{-/-}$ mice. Related to Figure 1. A summary of genes in the somatotroph axis and mitogenic signaling that are differentially expressed in the livers of SIRT7$^{-/-}$ mice compare to the wild type controls based on the microarray analyses. The listed p values are not corrected for multiple testing. Refer to Data S2 for complete analyses including multiple testing.

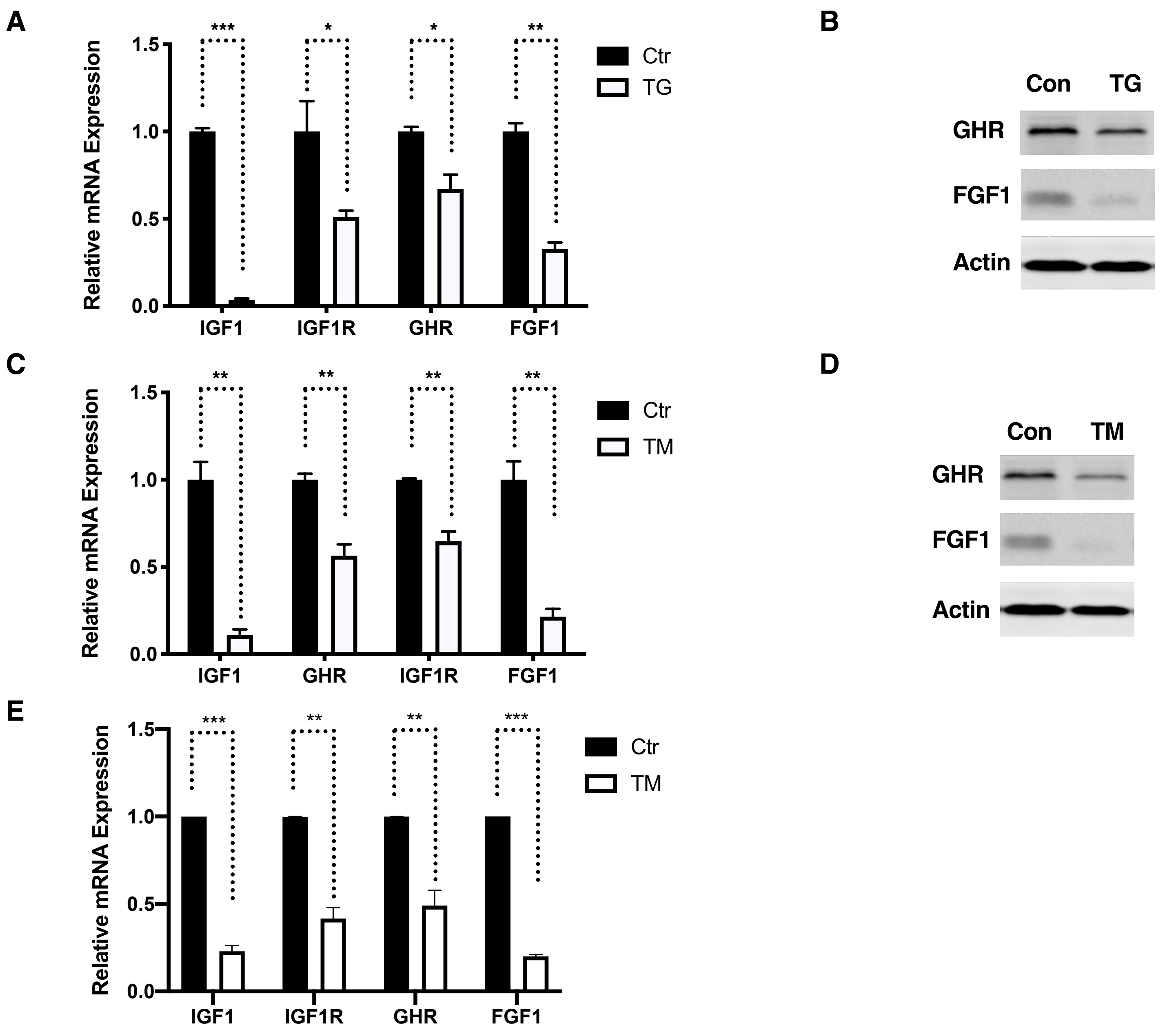

Figure S3. ER stress triggers the suppression of the somatotroph axis in hepatocytes. Related to Figure 2.
A-D, Quantitative real-time PCR and Western analyses of expression of indicated genes in Hepa 1-6 cells treated with thapsigargin (A, B) or tunicamycin (C, D). n=2.
E, Quantitative real-time PCR analyses of expression of indicated genes in primary hepatocytes treated with tunicamycin. n=2.
Error bars represent standard errors. * represents $p < 0.05$. ** represents $p < 0.01$. *** represents $p < 0.001$.

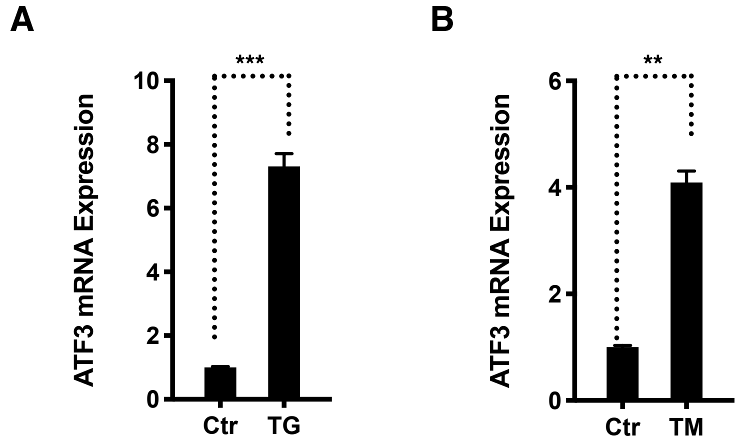

C

Gene Title	Gene Symbol	Binding Region
IGF1 Receptor	IGF1R	Enhancer
IGF2 mRNA Binding Protein 1	IGF2BP1	Promoter/Enhancer
IGF2 mRNA Binding Protein 3	IGF2BP3	Promoter/Enhancer
IGF2 Receptor	IGF2R	Promoter/Enhancer
IGF Binding Protein 3	IGFBP3	Promoter/Enhancer
IGF Binding Protein 4	IGFBP4	Promoter/Enhancer
IGF Binding Protein 6	IGFBP6	Promoter/Enhancer
IGF Like Family Member 4	IGFL4	Promoter/Enhancer
IGF Like Family Receptor 1	IGFLR1	Enhancer

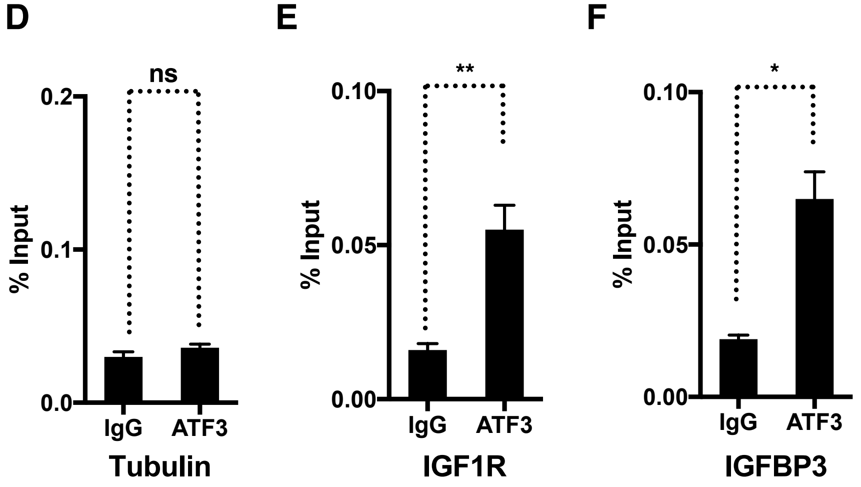

Figure S4. ATF3 is induced by protein folding stress and binds to the promotors or enhancers of IGF-related genes. Related to Figure 3.
A, B, Quantitative real time PCR analyses comparing the mRNA expression of ATF3 in Hepa 1-6 cells treated with or without ER stress inducers thapsigargin (A), tumicamycin (B). n=2-3.
C, A summary of IGF-related genes as ATF3 targets based on ChIP sequencing analyses using the Harmonizome web portal.
D-F, ChIP with ATF3 antibody followed by quantitative real-time PCR showing ATF3 occupancy at the promoters of the indicated genes in the mouse liver. n=2-3.
Error bars represent standard errors. * represents $p < 0.05$. ** represents $p < 0.01$. * represents $p < 0.001$. ns represents $p > 0.05$.**

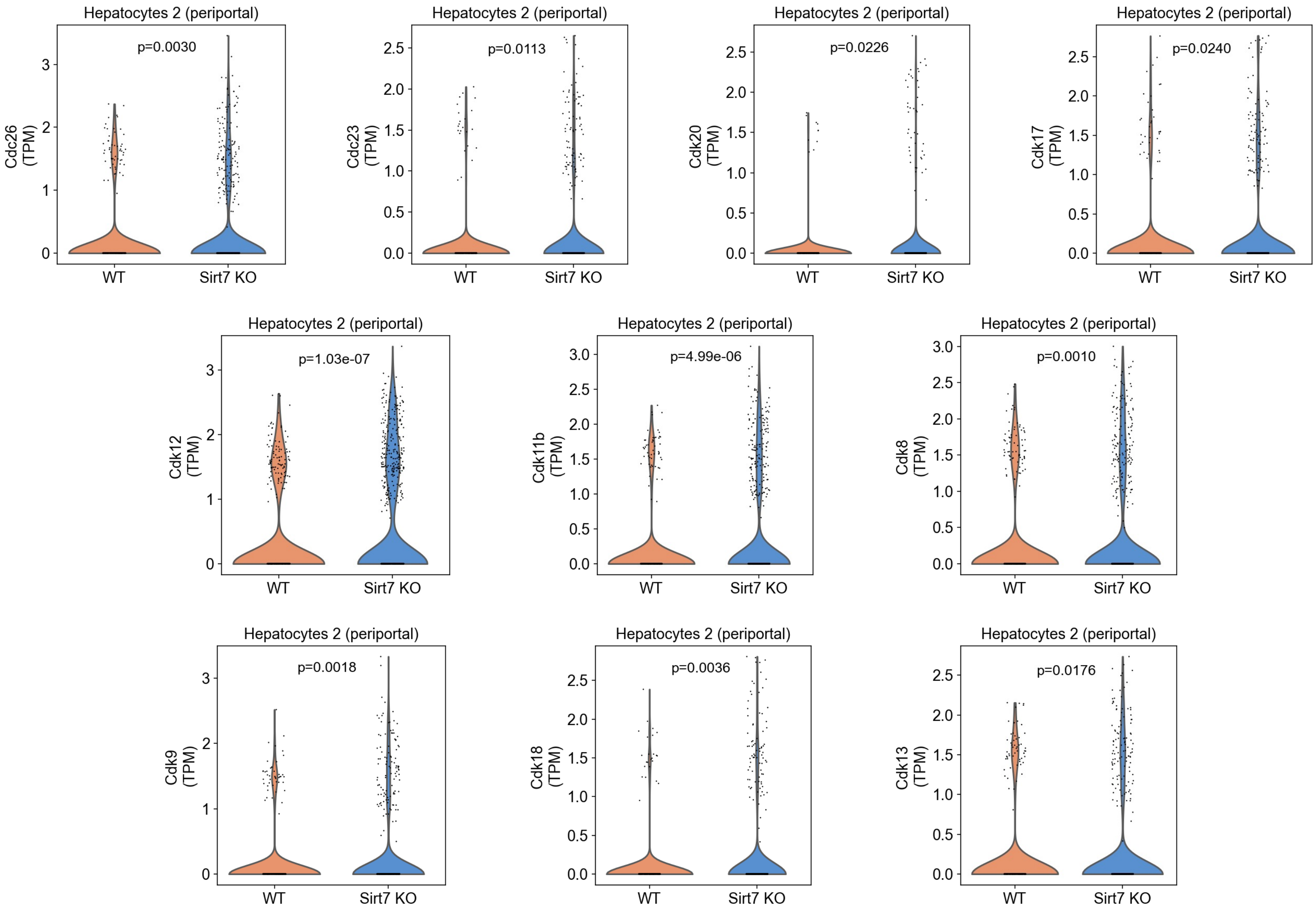

Figure S5. Increased expression of cell cycle genes in hepatocytes of the livers of SIRT7$^{-/-}$ mice. Related to Figure 4.

Violin plots showing the expression of representative cell cycle genes in hepatocytes of the livers of WT and SIRT7$^{-/-}$ mice. Each dot represents the gene expression levels in one cell. n=3 mice. P values are false discovery rate-corrected, MAST differential expression test.

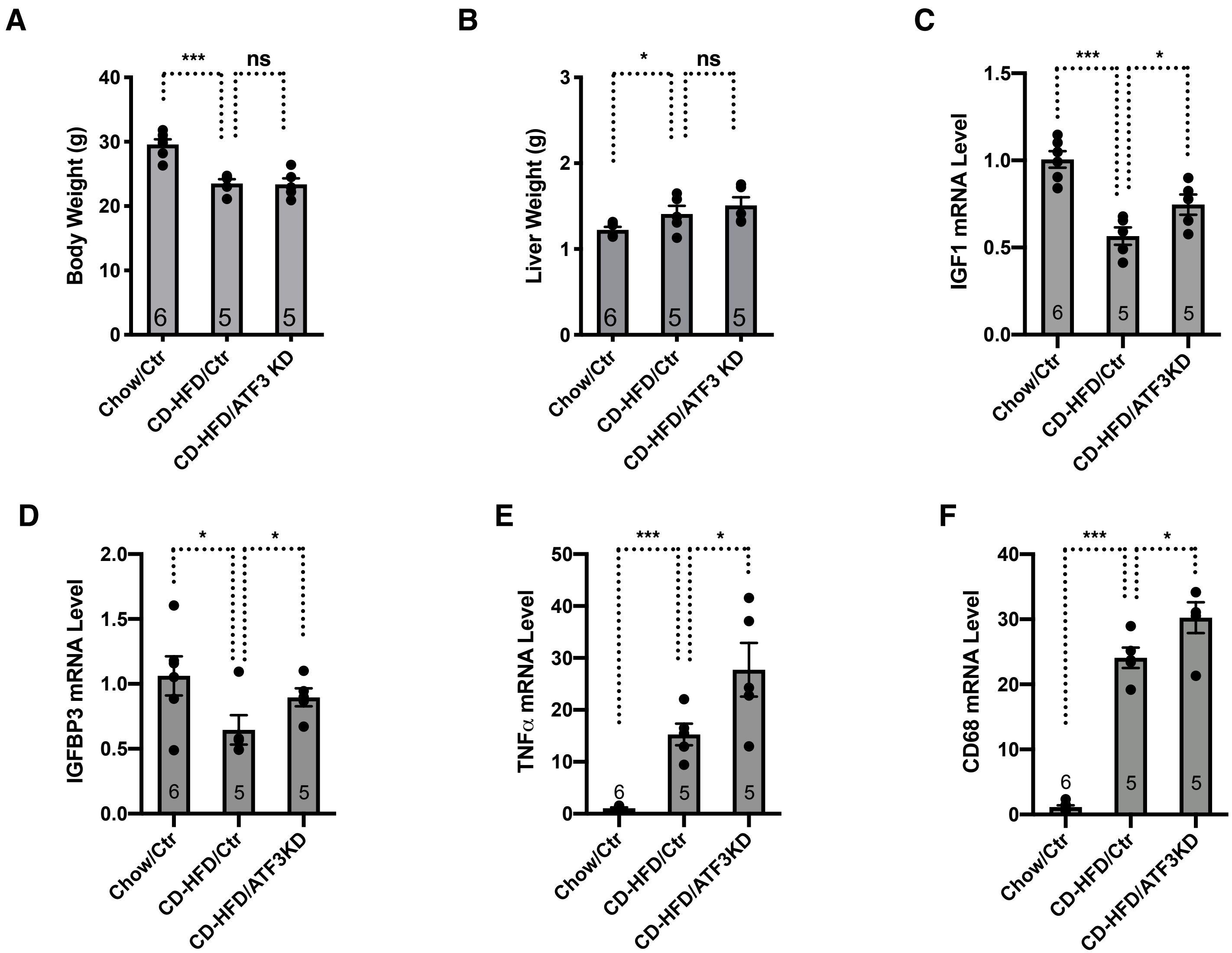

Figure S6. Suppression of the somatotroph axis controls liver damage in mice fed a CD-HFD. Related to Figure 5.
Comparison of wild type mice with or without ATF3 knockdown in the livers fed a chow diet or a CD-HFD for 8 weeks.
A, Body weight. n=5-6 mice.
B, Liver weight. n=5-6 mice
C-F, Quantitative real-time PCR analyses of expression of indicated genes in the livers. n=5-6 mice
Error bars represent standard errors. * represents $p < 0.05$. * represents $p < 0.001$. ns represents $p > 0.05$.**

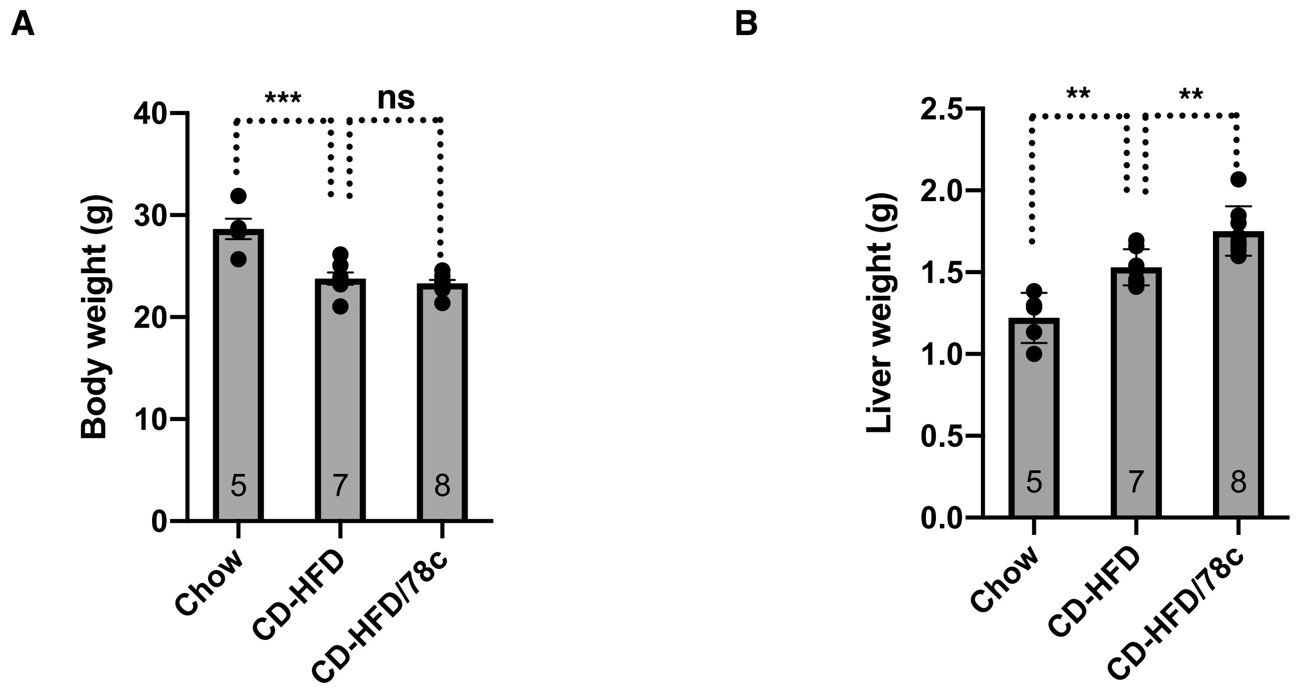

Figure S7. The effects of 78c on CD-HFD mice. Related to Figure 7.
Mice were fed a chow diet or a CD-HFD for 3 weeks before the treatment with or without 78c for 4 weeks. Data shown are body weight (A) and liver weight (B). n=5-8 mice
